# Screening, Mechanistic Investigation, and In Vivo Efficacy of a Compound Chinese Herbal Formulation Against Bovine-Derived *Escherichia coli* K99

**DOI:** 10.3390/life16101710

**Published:** 2026-10-12

**Authors:** Tianru Gao, Xiaoyu Zhang, Li Chen, Xuqiang Zhang, Yuntai Li, Yuxin Tan, Zhiqiang Zhang, Shaohua Wang, Qiumei Shi, Tonglei Wu

**Affiliations:** 1 Hebei Provincial Key Laboratory of Preventive Veterinary Medicine, Hebei Normal University of Science and Technology, Qinhuangdao 066004, China; 13131505031@163.com (T.G.); zxy1509433720@163.com (X.Z.); 18310985287@163.com (L.C.); zhangxuqiang020507@163.com (X.Z.); 18812098542@163.com (Y.L.); taxlnjsg@126.com (Y.T.); zhangzhiqiang87@hevttc.edu.cn (Z.Z.); 2 College of Animal Science and Technology, Hebei North University, Zhangjiakou 075000, China

**Keywords:** *Escherichia coli*, K99, compound herbal formula, antibacterial mechanism, safety evaluation

## Abstract

**Background:** *Escherichia coli (E. coli)* K99 is one of the major pathogens responsible for neonatal calf diarrhea, characterized by widespread transmission and strong pathogenicity, leading to high mortality in calves and substantial economic losses in the cattle industry. Antibiotic-based therapies are increasingly challenged by drug residues and continuous emergence of antimicrobial resistance, which creates an urgent demand for safe and effective alternative antimicrobial agents. Chinese herbal medicines (CHMs), featuring low toxicity, abundant resources and synergistic effects of multiple bioactive constituents, have become promising candidates for new antibacterial agents. **Objectives:** This study aimed to screen Chinese herbal medicines for antibacterial activity against bovine-derived *E. coli* K99-2, develop a compound herbal formula F1 following the traditional “Jun-Chen-Zuo-Shi” compatibility principle, and systematically characterize its chemical composition, antibacterial mechanism, in-vivo safety and protective therapeutic efficacy. **Materials and Methods:** Seventy Chinese herbal medicines were subjected to in-vitro antibacterial screening using the Oxford cup agar diffusion assay. Candidate compound formulas were designed according to screening outcomes and “Jun-Chen-Zuo-Shi” theory. The MIC and MBC of the optimized formula F1 were determined via broth microdilution. LC-MS was performed to profile chemical constituents of F1. Growth curves, membrane potential, cell-wall permeability, intracellular protein and ATP levels, gel retardation assay and scanning electron microscopy were applied to investigate antibacterial mechanisms. A 28-day repeated oral gavage toxicity test in Kunming mice was conducted to evaluate biosafety. The LD_50_ of *E. coli* K99-2 was determined by intraperitoneal challenge, and a mouse infection model was used to assess the in-vivo protective effect of F1. **Results:** *Fructus Mume* and *Cornus officinalis* displayed prominent antibacterial activity against K99-2. The optimized compound formula F1 produced an inhibition-zone diameter of 21 mm; both MIC and MBC were 62.5 mg/mL. Putatively annotated constituents of F1 included furan derivatives, amino acids, flavonoids, polyphenolic compounds and organic acids. F1 triggered bacterial cell-envelope damage, membrane depolarization, reduction of intracellular protein content and disturbance of ATP-associated energy metabolism, while it did not form stable complexes with bacterial genomic DNA. The 28-day oral toxicity assay revealed no obvious toxic manifestations within the tested dose range in mice. In the K99-2 infection-challenge experiment, oral administration of F1 raised the mouse survival rate from 40% (untreated infected control) to 73%. **Discussion:** Compound herbal formula F1 exerts multi-target antibacterial activity against *E. coli* K99-2 mainly by disrupting bacterial structural integrity and interfering with energy metabolism rather than directly binding to genomic DNA. F1 demonstrates favorable preliminary biosafety and can improve survival of K99-2-infected mice, indicating its potential as a veterinary herbal candidate for prevention and control of calf diarrhea caused by *E. coli* K99. Further work is required to identify key bioactive components, optimize dosage and verify efficacy in calf clinical settings. This research provides experimental basis for developing antimicrobial herbal veterinary preparations.

## 1. Introduction

Antimicrobial resistance is becoming increasingly serious and poses a major challenge to the prevention and treatment of bacterial diarrhea in calves. *Escherichia coli* (*E. coli*) K99 is an important pathogen associated with neonatal calf diarrhea and can cause severe dehydration and even death in calves [1,2]. K99, also known as the F5 fimbrial antigen, is an important adhesin associated with enterotoxigenic *E. coli* (ETEC) and facilitates bacterial attachment to specific receptors on intestinal epithelial cells, thereby promoting colonization of the small intestine [3]. Following intestinal colonization, enterotoxigenic strains may produce enterotoxins, particularly the heat-stable enterotoxin STa, which disrupts intestinal fluid and electrolyte homeostasis and contributes to secretory diarrhea [4]. Thus, the pathogenesis of K99-associated neonatal calf diarrhea involves both fimbrial adhesion and enterotoxin-mediated intestinal dysfunction. Antibiotics remain the main approach for controlling bacterial infections; however, their inappropriate use can promote the emergence and spread of antimicrobial-resistant bacteria [5,6]. Shi et al. [7] reported that the abundance and diversity of antibiotic resistance genes were significantly higher in the intestinal microbiota of diarrheic calves than in healthy calves. Other studies have also shown that calf-derived *E. coli* isolates are resistant to multiple commonly used antimicrobial agents and often exhibit multidrug-resistant phenotypes [8,9]. The increasing prevalence of antimicrobial resistance not only limits treatment options but also poses potential public health risks through the transmission of resistant bacteria and resistance genes [5,6]. Therefore, the development of safe and effective antimicrobial agents as potential alternatives or complementary approaches to conventional antibiotics is of great importance.

Chinese herbal medicines contain various chemical constituents, including alkaloids, flavonoids, and organic acids, and some of these compounds have demonstrated antimicrobial potential [10]. Previous studies have shown that herbal extracts and some of their components exhibit inhibitory effects against *E. coli*, *Staphylococcus aureus*, and other pathogenic bacteria [10,11,12]. Their antibacterial activities have been associated with several potential mechanisms, including alteration of bacterial membrane structure and function, interference with macromolecular synthesis, and disruption of energy metabolism [13,14]. However, the safety profiles of herbal medicines can vary considerably among different plant species and may be affected by their chemical composition, dosage, and administration conditions [15]. Therefore, the safety and potential toxicity of herbal preparations should be carefully evaluated according to their specific composition and administration conditions. In recent years, research in this field has gradually expanded from the evaluation of individual herbs to the investigation of herbal formulas and combination therapies. Ding et al. [11] found that a Chinese herbal formula combined with Microcin J25 exhibited enhanced antibacterial activity against multidrug-resistant *E. coli* isolated from calves and improved diarrhea symptoms and intestinal microbial balance in calves. Qin et al. [12] summarized recent progress in the combined application of traditional Chinese medicine and antibiotics against bacterial infections, providing further evidence for the potential antimicrobial applications of Chinese herbal medicines.

Although existing studies support the antibacterial potential of Chinese herbal medicines, studies specifically targeting bovine-derived *E. coli* K99 that integrate herbal screening, formula development, chemical profiling, and both in vitro and in vivo evaluation remain limited. The effects of herbal formulas on bacterial structures and physiological functions, as well as whether their antibacterial activity observed in vitro can provide protective effects in vivo, require further investigation.

In this study, 70 selected Chinese medicinal materials were screened for antibacterial activity against the bovine-derived *E. coli* K99-2 strain, and the Chinese herbal formula F1 was developed based on antibacterial activity and the traditional principle of “Jun-Chen-Zuo-Shi” compatibility. Liquid chromatography–mass spectrometry (LC-MS) was used to characterize the chemical composition of F1. The antibacterial effects of F1 were further investigated by evaluating bacterial growth, cell envelope integrity, energy metabolism, and interactions with genomic DNA. In addition, a 28-day repeated oral administration study and an intraperitoneal (i.p.) infection challenge experiment in mice were conducted to evaluate the preliminary safety and in vivo protective effects of F1. We hypothesized that F1 may exert antibacterial effects by affecting bacterial cell envelope integrity and energy metabolism, and may provide protective effects against *E. coli* K99 infection in mice. This study aimed to characterize the antibacterial effects of F1 and explore its possible antibacterial mechanisms, providing experimental evidence for the development of candidate veterinary herbal preparations.

## 2. Materials and Methods

### 2.1. Chinese Herbal Medicines, Experimental Mice, and E. coli K99

A total of 70 Chinese herbal medicines were purchased from Hebei Tangren Pharmaceutical Co., Ltd. (Tangshan, China). All materials were commercially available Chinese herbal medicine pieces that met the quality standards of the *Chinese Pharmacopoeia*. They were stored in sealed containers at room temperature in a dry and dark place until use. The names, Latin names, and medicinal parts are listed in Table 1.

Female Kunming mice (5–6 weeks old) were purchased from Beijing SPF Biotechnology Co., Ltd. (Beijing, China). After arrival, mice were acclimatized for 7 days before the experiments. Mice were maintained under specific pathogen-free (SPF) conditions at 22.0 ± 0.5 °C with 60 ± 10% relative humidity and a 12 h light/dark cycle. Mice were housed five per cage and provided with free access to standard laboratory chow and water ad libitum throughout the experimental period.

The *E. coli* K99 strain used in this study was isolated from anal swab samples of diarrheic calves collected from a large-scale beef cattle farm in Tangshan City, Hebei Province. The isolated strain was identified as K99-2 and stored at the Key Laboratory of Preventive Veterinary Medicine of Hebei Province.

### 2.2. In Vitro Antibacterial Activity of Single Chinese Herbal Medicines Against K99-2

Ten grams of each single Chinese herbal medicine was mixed with 100 mL of deionized water and soaked at room temperature for 12 h. After soaking, the herbal materials were transferred to a round-bottom flask and heated to boiling for 10 min, followed by extraction at a gentle boil for 45 min. The decoction was then filtered through gauze, and the remaining herbal residues were extracted twice more using the same procedure. The three decoctions were combined and centrifuged, and the supernatant was collected. The supernatant was gently concentrated to a final volume of 10 mL to prepare the aqueous herbal extracts. The extracts were sterilized by autoclaving and stored for subsequent experiments.

The antibacterial activity of the aqueous herbal extracts against K99-2 was evaluated using the Oxford cup agar diffusion method. Briefly, 100 μL of the standardized K99-2 bacterial suspension (1 × 10^7^ CFU/mL) was evenly spread onto Mueller–Hinton (MH) agar plates. Sterile Oxford cups were placed vertically on the agar surface, and 200 μL of each herbal extract was added to the cups. The plates were incubated at 37 °C for 14–18 h, after which the diameter of the inhibition zone was measured to evaluate antibacterial activity. According to a previously reported method for evaluating the sensitivity of *E. coli* to plant-derived active extracts [16], antibacterial activity was classified according to the inhibition zone diameter. In this study, inhibition zone diameters of >20 mm, 15–20 mm, 10–15 mm, and <10 mm were classified as extremely sensitive, highly sensitive, moderately sensitive, and slightly sensitive, respectively.

### 2.3. In Vitro Antibacterial Activity of Compound Herbal Formulas Against K99-2

Based on the in vitro antibacterial screening of individual Chinese herbs, candidate formulas were developed according to the antibacterial activity of each herb against bovine-derived *Escherichia coli* K99-2, while considering traditional Chinese medicine compatibility, contraindications and the “Jun-Chen-Zuo-Shi” principle. During formula development, herbs with relatively strong antibacterial activity and a major contribution to the overall antibacterial effect were selected as Jun herbs. Herbs with good antibacterial activity that could support or enhance the effects of the Jun herbs were selected as Chen herbs. Herbs that could provide auxiliary antibacterial effects, regulate the overall properties of the formula, or improve its overall efficacy were used as Zuo herbs. Finally, herbs with a harmonizing role were included as Shi herbs. On this basis, three candidate formulas, F1, F2, and F3, were developed by adjusting the combinations and proportions of the major herbs to compare the effects of different compatibility patterns on antibacterial activity against K99-2. The compositions and proportions of the three formulas are shown in Table 2.

Aqueous extracts of the compound herbal formulas were prepared using the same method as described for single herbal extracts. Their antibacterial activities were evaluated using the same in vitro antibacterial assay. In addition, according to the guidelines of the Clinical and Laboratory Standards Institute (CLSI, 2007) [17] with appropriate modifications, the minimum inhibitory concentration (MIC) and minimum bactericidal concentration (MBC) of the compound herbal extracts against K99-2 were determined using the broth microdilution method.

Briefly, the K99-2 bacterial suspension was diluted with LB broth to 1 × 10^6^ CFU/mL. A volume of 100 μL LB broth was added to wells 1–10 of a 96-well plate. Then, 100 μL of the compound herbal extract was added to the first well, mixed thoroughly, and serially two-fold diluted to the tenth well. After mixing, 100 μL of excess solution was removed from the tenth well. Subsequently, 100 μL of K99-2 bacterial suspension (1 × 10^6^ CFU/mL) was added to each experimental well and mixed thoroughly. The eleventh well containing the herbal extract served as the negative control, while the twelfth well containing an equal volume of bacterial suspension served as the positive growth control.

The 96-well plates were incubated at 37 °C for 18 h. The lowest concentration without visible bacterial growth was recorded as the MIC. Bacterial suspensions from wells with concentrations equal to or higher than the MIC were streaked onto agar plates and incubated at 37 °C for 18 h. The lowest concentration showing no colony formation was defined as the MBC.

### 2.4. Liquid Chromatography–Mass Spectrometry Analysis of Active Compounds in Compound Herbal Formula F1

A 100 μL aliquot of the aqueous extract of compound herbal formula F1 was mixed with 400 μL methanol. The mixture was vortexed and centrifuged at 13,000× *g* for 10 min. The supernatant was collected, filtered through a 0.22 μm membrane, and subjected to liquid chromatography–mass spectrometry (LC-MS) analysis. The LC-MS analysis was conducted following the procedure reported by Yang et al. [18], with minor modifications to accommodate the experimental conditions.

LC-MS analysis was performed by Chengdu Century Meiyang & Technology Co., Ltd. (Chengdu, China). Chromatographic separation was conducted using a Thermo Fisher Scientific Vanquish UHPLC system (Waltham, MA, USA) equipped with an Agilent ZORBAX SB-C18 column (2.1 mm × 100 mm; Agilent Technologies, Santa Clara, CA, USA). The column temperature was maintained at 40 °C, with an injection volume of 2 μL and a flow rate of 0.3 mL/min. Mobile phase A consisted of 0.1% formic acid in water, and mobile phase B was acetonitrile. The gradient elution program was as follows: 0–3 min, 2% B; 3–25 min, B increased linearly from 2% to 98%; 25–27 min, maintained at 98% B for isocratic elution; and 27–30 min, B decreased from 98% to 2%, followed by equilibration with 2% B.

Mass spectrometry analysis was performed using a Thermo Fisher Scientific Q Exactive Plus mass spectrometer (Waltham, MA, USA) equipped with an electrospray ionization (ESI) source. The spray voltage was set at 3.5 kV, the ion transfer tube temperature at 350 °C, and the vaporizer temperature at 300 °C. The sheath gas and auxiliary gas were set at 35 and 10 arbitrary units (arb), respectively. Data were acquired in both positive and negative ion modes using high-resolution full-scan analysis combined with data-dependent acquisition tandem mass spectrometry (DDA-MS/MS). The mass range for MS1 scanning was *m*/*z* 100–1500, the initial fragment ion range for MS2 was *m*/*z* 50, and the collision energy was set at 40 eV.

Quality control (QC) samples were prepared from the same batch of F1 aqueous extract using the identical preparation procedure. QC samples were periodically inserted into the analytical sequence before and during sample detection to evaluate retention time stability, mass deviation, and peak area reproducibility. Methanol was used as a solvent blank to eliminate interference from solvent and system background signals.

Compound annotation was performed by matching retention time (Rt), accurate observed *m*/*z*, adduct ion information, MS/MS fragmentation patterns, and spectral information against chemical databases. Candidate compounds were further screened using a mass error of |ΔMass| < 5 ppm and an mzCloud spectral matching confidence ≥ 80%. Because no authentic reference standards were used in the present study, the identified compounds were considered putatively annotated candidates rather than definitively confirmed compounds. Further confirmation using authentic reference standards, including comparison of retention times and MS/MS fragmentation patterns, will be performed in future studies. Peak area values were used only for semi-quantitative comparison of the relative abundances of the detected candidate compounds and were not interpreted as absolute concentrations, because ionization efficiencies and detector responses may vary among different compounds [19].

### 2.5. Effect of Compound Herbal Formula F1 on the Growth Curve of E. coli K99-2

A single colony of *E. coli* K99-2 was inoculated into LB broth and cultured at 37 °C with shaking at 180 rpm for 12 h. The bacterial suspension was then transferred into LB broth containing different concentrations of compound herbal formula F1, resulting in final F1 concentrations of 1/4 × MIC, 1/2 × MIC, and 1 × MIC. LB broth without F1 was used as the untreated control. Equal volumes of the bacterial culture were inoculated into each treatment group, and the initial OD600 values at 0 h were approximately 0.4 across all groups.

All groups were cultured in a 37 °C shaking incubator for 12 h. Samples (300 μL) were collected every 2 h, and the OD_600nm_ values were measured using a microplate reader. The growth curve shown represents a single experimental measurement; therefore, variability among independent replicates was not assessed. The growth curve assay was conducted following the method reported by Wiegand et al. [20], with minor modifications to adapt to the experimental conditions.

### 2.6. Effect of Compound Herbal Formula F1 on the Membrane Potential of K99-2 Cells

The membrane potential of K99-2 cells was determined using the rhodamine fluorescence method [21] with appropriate modifications. Briefly, K99-2 cells were cultured in LB broth until the logarithmic growth phase. Two F1 treatment groups were established with final concentrations of 1 × MIC and 2 × MIC, while LB broth without F1 was used as the blank control. All groups were incubated in a 37 °C shaking incubator for 2 h, and 1 mL of bacterial suspension was collected every hour for analysis.

The collected bacterial cells were harvested by centrifugation, washed, and resuspended three times with phosphate-buffered saline (PBS). Rhodamine 123 was then added to a final concentration of 2 μg/mL, followed by incubation in the dark for 30 min. After incubation, the cells were washed and resuspended again with PBS. Fluorescence intensity was measured immediately after resuspension using a multifunctional microplate reader with excitation and emission wavelengths of 475 nm and 525 nm, respectively. Changes in the membrane potential of K99-2 cells were evaluated based on the mean fluorescence intensity (MFI).

### 2.7. Effect of Compound Herbal Formula F1 on Cell Wall Permeability of K99-2

The effect of compound F1 on cell wall integrity of K99-2 was evaluated by measuring alkaline phosphatase (AKP) activity [22]. K99-2 cells were inoculated into LB broth and cultured at 37 °C until reaching the logarithmic growth phase. The experiment included a compound F1-treated group (final concentration: 1 × MIC) and an untreated control group without compound F1 supplementation. Both groups were incubated in a shaking incubator at 37 °C for 5 h, and 1 mL of bacterial suspension was collected every hour. The samples were centrifuged to remove bacterial cells, and the supernatants were collected. Subsequently, extracellular AKP activity was determined using an AKP assay kit (P0321S, Beyotime Biotechnology, Shanghai, China) according to the manufacturer’s instructions to evaluate changes in cell wall permeability of K99-2 induced by compound F1.

### 2.8. Effect of Compound Herbal Formula F1 on Intracellular Protein Content of K99-2

K99-2 cells were cultured in LB broth until the logarithmic growth phase. F1 was added to the cultures at final concentrations of 1 × MIC and 2 × MIC, while cultures without F1 treatment served as the control group. The cultures were incubated at 37 °C in a shaking incubator, and bacterial samples were collected at 0, 2, 4, and 6 h. At each time point, bacterial cells were harvested by centrifugation and washed three times with 1 × PBS buffer.

The bacterial cells were resuspended, and the OD_600nm_ value of the suspension was adjusted to 2.0. A 1 mL aliquot of K99-2 suspension was subjected to ultrasonic disruption on ice (300 W, pulse interval 1.1 s) for 10 min. The resulting cell lysate was collected, and the intracellular protein content was determined using a BCA protein assay kit (AR0146, Bioss Biotechnology Co., Ltd., Wuhan, China) according to the manufacturer’s instructions. All experiments were performed with three independent replicates. The extraction and quantification of intracellular proteins were conducted according to the method reported by Hao et al. [23], with minor modifications to accommodate the experimental conditions.

### 2.9. Effect of Compound Herbal Formula F1 on Intracellular ATP Levels in K99-2

K99-2 cells were cultured in LB broth until the logarithmic growth phase. F1 was added at final concentrations of 1 × MIC and 2 × MIC, and the cultures were incubated at 37 °C in a shaking incubator. Samples were collected at 0, 2, and 4 h. At each time point, bacterial cells were collected by centrifugation, washed three times with 1 × PBS, and resuspended.

A 1 mL aliquot of K99-2 suspension was disrupted by ultrasonic treatment on ice (300 W, pulse interval 1.1 s) for 10 min. The disrupted suspension was centrifuged at 8000 rpm for 10 min using a refrigerated centrifuge, and the supernatant was collected. The ATP content was measured using an ATP assay kit (BC0300, Solarbio Science & Technology Co., Ltd., Beijing, China) according to the manufacturer’s instructions. The experiment was repeated three times, and the average value was calculated. K99-2 cultures without F1 treatment were used as the blank control. The intracellular ATP extraction and quantification were performed according to the method described by Xu et al. [24], with minor modifications based on the experimental conditions.

### 2.10. Effect of Compound Herbal Formula F1 on the Genomic DNA of K99-2

Genomic DNA of K99-2 was extracted using a bacterial genomic DNA extraction kit (D1600, Solarbio Science & Technology Co., Ltd., Beijing, China) according to the manufacturer’s instructions. Based on the method described by Sousa et al. [25] with appropriate modifications, a gel retardation assay was performed to evaluate the interaction between F1 and K99-2 genomic DNA.

Briefly, 5 μL of K99-2 genomic DNA solution was mixed with 5 μL of F1 at different concentrations, resulting in final concentrations of 1/2 × MIC, 1 × MIC, 2 × MIC, and 4 × MIC. A blank control group containing 5 μL ddH_2_O was included. The reaction volume was adjusted to 20 μL with ddH_2_O and mixed thoroughly, followed by incubation at 37 °C for 1 h.

After incubation, samples were subjected to 1% agarose gel electrophoresis. The gels were visualized using a gel imaging system, and the migration retardation of K99-2 genomic DNA after treatment with different concentrations of F1 was analyzed.

### 2.11. Scanning Electron Microscopy Observation of Ultrastructural Changes in K99-2 Cells Treated with Compound Herbal Formula F1

K99-2 cells in the logarithmic growth phase were treated with compound herbal formula F1 at a final concentration of 1 × MIC. After thorough mixing, the cultures were incubated at 37 °C for 4 h. Untreated K99-2 cells were cultured in parallel under the same conditions and served as the control group. The bacterial cells were collected by centrifugation at 5000 rpm (approximately 2290× *g*) for 10 min and fixed in 2.5% glutaraldehyde for 12 h. After fixation, the fixative was removed, and the samples were immersed in PBS buffer for 6 h. The samples were subsequently dehydrated through a graded ethanol series of 30%, 50%, 70%, 80%, 95%, and 100%, with each dehydration step performed for 10 min, followed by treatment with isoamyl acetate for 15 min. The samples were then dried using a critical-point dryer (EM CPD300, Leica Microsystems, Wetzlar, Germany), mounted on conductive adhesive, and sputter-coated with gold for 30 s using an EM ACE200 sputter coater (Leica Microsystems, Wetzlar, Germany).

Finally, the samples were examined and imaged using a GEMINI 560 scanning electron microscope (ZEISS, Oberkochen, Germany) at an accelerating voltage of 3.00 kV and a working distance of 9.3 mm using the SE2 detector. Representative images for morphological observation were acquired at 20,000× magnification. For quantitative morphological analysis, five independent fields of view per group were selected at 5000× magnification. Cells exhibiting obvious shrinkage, deformation, collapse, surface depression, rupture, or fragmentation were classified as morphologically damaged cells. Total and damaged cells in each field were manually counted using Fiji (ImageJ 1.54p, accessed on 21 September 2026), and the percentage of damaged cells was calculated as follows:

Damaged cells (%) = (damaged cells/total cells) × 100

The control and F1-treated samples were harvested, fixed, and processed in parallel under identical conditions.

### 2.12. 28-Day Oral Toxicity Study

Thirty 6-week-old Kunming mice were randomly divided into six groups (groups A, B, C, D, E, and F), with five mice in each group. Only female mice were used in the present study. Mice in groups A–E were orally administered 200 μL of F1 solution at different concentrations, with final concentrations of 62.5, 125, 250, 600, and 1000 mg/mL, expressed as raw herb equivalents, corresponding to approximately 625, 1250, 2500, 6000, and 10,000 mg/kg body weight/day, respectively, based on an average mouse body weight of 20 g. The F1 preparations were readily administered through the gavage needle without apparent viscosity-related difficulty. Mice in group F received an equal volume of 1 × PBS solution as the control. The treatment was continued for 28 consecutive days. During the experimental period, clinical signs, general condition, and survival status were monitored and recorded daily. The 28-day repeated-dose oral toxicity study was performed according to the method described by Bak et al. [26], with minor modifications based on the experimental conditions. The limited sample size and use of only female mice should be considered when interpreting the safety findings; future studies will include larger cohorts and both sexes.

### 2.13. Hematological and Blood Biochemical Parameters

After 28 days of continuous oral administration, mice were fasted for more than 12 h, and blood samples were collected from the tail tip. All experimental groups underwent the same fasting procedure before blood collection to minimize potential variations caused by feeding status. A portion of anticoagulated whole blood collected using K2-EDTA as the anticoagulant was used for routine blood analysis using an automated hematology analyzer (TEK-VET3, Jiangxi Tecom Science and Technology Co., Ltd., Nanchang, China), while the remaining blood samples were processed to obtain serum. Serum was separated by centrifugation at 1500× *g* for 10 min at room temperature. Serum samples were submitted to Jiangsu Sulianke Biotechnology Co., Ltd. (Yancheng, China) for biochemical analysis using an automated biochemical analyzer (BK-280, Shandong Biobase Biotechnology Co., Ltd., Jinan, China). The results were expressed as mean ± standard deviation (SD).

### 2.14. Organ Index Measurement

At the end of the experiment, mice from each group were weighed after fasting, and the terminal fasting body weight was recorded, and then euthanized by CO_2_ inhalation prior to organ collection. The mice were then dissected, and the organs were collected. The heart, liver, spleen, lungs, and kidneys were first examined visually, and gross morphological changes were recorded. The organs were then gently rinsed with sterile saline to remove surface residues, dried with filter paper, and weighed. For paired organs, including the lungs and kidneys, both sides were collected and weighed together to determine the total organ mass. The organ index was calculated using the following formula:

Organ index (%) = (organ weight/terminal fasting body weight) × 100%

The organ index measurement was performed according to the method described by Xu et al. [27], with minor modifications based on the experimental conditions.

### 2.15. Histopathological Examination of Ileum Tissue

Ileum tissue samples were collected from mice at the end of the experiment for histological analysis. Briefly, approximately 1 cm of ileum tissue was collected and immediately washed gently with precooled saline to remove surface residues. The tissues were then fixed in precooled 4% paraformaldehyde solution for 24 h. After fixation, samples were dehydrated using a graded ethanol series (70–100%), cleared in xylene for 30 min, and embedded in paraffin. The paraffin-embedded tissues were sectioned into 4–6 μm slices, stained with hematoxylin and eosin (H&E), and finally observed under an Olympus BX51T-PHD-J11 light microscope equipped with an Olympus CMOS camera (Olympus Corporation, Tokyo, Japan) to evaluate pathological changes in the ileum tissue. Histological images were acquired and analyzed using Image-Pro Plus 6.0 software (Media Cybernetics, Rockville, MD, USA; accessed on 23 September 2026), and representative images were obtained at 200× magnification. Histopathological lesions were evaluated using a semi-quantitative scoring system based on mucosal damage, villus injury, inflammatory cell infiltration, and epithelial integrity. The stained sections were independently assessed by two researchers who were blinded to the experimental groups. Histopathological examination of ileum tissue was performed according to the method described by Fischer et al. [28], with minor modifications based on the experimental conditions.

### 2.16. Determination of LD_50_ of K99-2

Thirty 6-week-old Kunming mice were randomly divided into six groups, with five mice per group. Although *E. coli* K99 is primarily associated with neonatal calf diarrhea through intestinal colonization and enterotoxin production, the intraperitoneal (i.p.) infection model used in this study was established to evaluate the acute systemic infection characteristics of K99-2 and was not intended to directly reproduce intestinal colonization or diarrhea in calves. Based on the preliminary experimental results, mice in the first five groups were intraperitoneally injected with 200 μL of K99-2 bacterial suspensions at different concentrations, ranging from 5.5 × 10^4^ to 5.5 × 10^8^ CFU per mouse. Mice in the sixth group received an equal volume of 1 × PBS solution as the blank control. The mice were continuously monitored for 14 days, and clinical symptoms and mortality were recorded. The median lethal dose (LD_50_) was calculated using the Karber method [29] according to the following formula:

log LD_50_ = Xm − i (∑p − 0.5) (1)

where Xm represents the logarithm of the highest dose, i represents the difference between the logarithms of two adjacent doses, and p represents the mortality rate of each group.

### 2.17. Effect of Compound Herbal Formula F1 on Mortality in K99-2-Infected Mice

Forty-five 6-week-old Kunming mice were randomly divided into three groups, with 15 mice in each group. Mice in groups A and B were intraperitoneally challenged with K99-2 bacterial suspension at the LD_50_ dose, while mice in group C received an equal volume of 1 × PBS solution as the blank control.

Six hours after infection, mice in group A were orally administered 200 μL of compound herbal formula F1 solution (1000 mg/mL) once every 24 h for 7 consecutive days. The initiation of treatment at 6 h post-infection was designed as an early post-infection intervention strategy to evaluate the protective effect of F1 against K99-2 infection. Mice in groups B and C received an equal volume of 1 × PBS solution by gavage. During the experimental period, clinical symptoms and survival status of mice in each group were continuously monitored and recorded.

### 2.18. Statistical Analysis

All statistical analyses were performed using GraphPad Prism version 8.0.2 (accessed on 19 September 2026). Data are presented as mean ± standard deviation (SD). Comparisons among multiple groups were performed using one-way analysis of variance (ANOVA) followed by Tukey’s multiple comparisons test. Time-course experiments, including membrane potential changes, AKP activity, intracellular protein content, and ATP content assays, were analyzed using two-way repeated measures ANOVA with Geisser–Greenhouse correction followed by Sidak’s multiple comparisons test. Survival curves were generated using the Kaplan–Meier method and compared using the Log-rank (Mantel–Cox) test. A *p*-value < 0.05 was considered statistically significant.

## 3. Results

### 3.1. Determination of Inhibition Zone Diameter of Single Chinese Herbal Medicines Against K99-2 In Vitro

In this study, the antibacterial activities of 70 Chinese herbal medicines against the K99-2 strain were evaluated in vitro. As shown in Table 3, a total of 20 herbal medicines exhibited antibacterial activity to varying degrees. According to the inhibition zone diameter criteria [16], five herbal medicines showed highly sensitive antibacterial effects, eight exhibited moderately sensitive effects, and seven showed low sensitivity. It should be noted that this assay was performed as a preliminary screening of the antibacterial activity of individual Chinese herbal medicines. Independent biological replicates were not conducted; therefore, the inhibition zone diameters are presented as individual measurements rather than Mean ± SD.

### 3.2. Determination of Inhibition Zone Diameter of Compound Herbal Formulas Against K99-2 In Vitro

The antibacterial activities of three compound herbal formulas (F1, F2, and F3) against the K99-2 strain were further evaluated, and the results are shown in Table 4. All three formulas exhibited antibacterial activity to some extent. The inhibition zone diameters of F1, F2, and F3 were 21, 19, and 19 mm, respectively. According to the inhibition-zone-based sensitivity criteria [16], F1 was classified as extremely sensitive within this classification system.

### 3.3. Determination of MIC and MBC of Compound Herbal Formula F1

As shown in Table 5, all three compound herbal formulas exhibited antibacterial activity against the K99-2 strain to varying degrees. The MIC and MBC values of F1 were both 62.5 mg/mL, whereas those of F2 and F3 were both 125 mg/mL. Based on these measured MIC and MBC values, subsequent experiments focused on the antibacterial mechanism and potential clinical application of F1.

### 3.4. Liquid Chromatography–Mass Spectrometry Analysis of Active Components in Compound Herbal Formula F1

**Figure 1:**
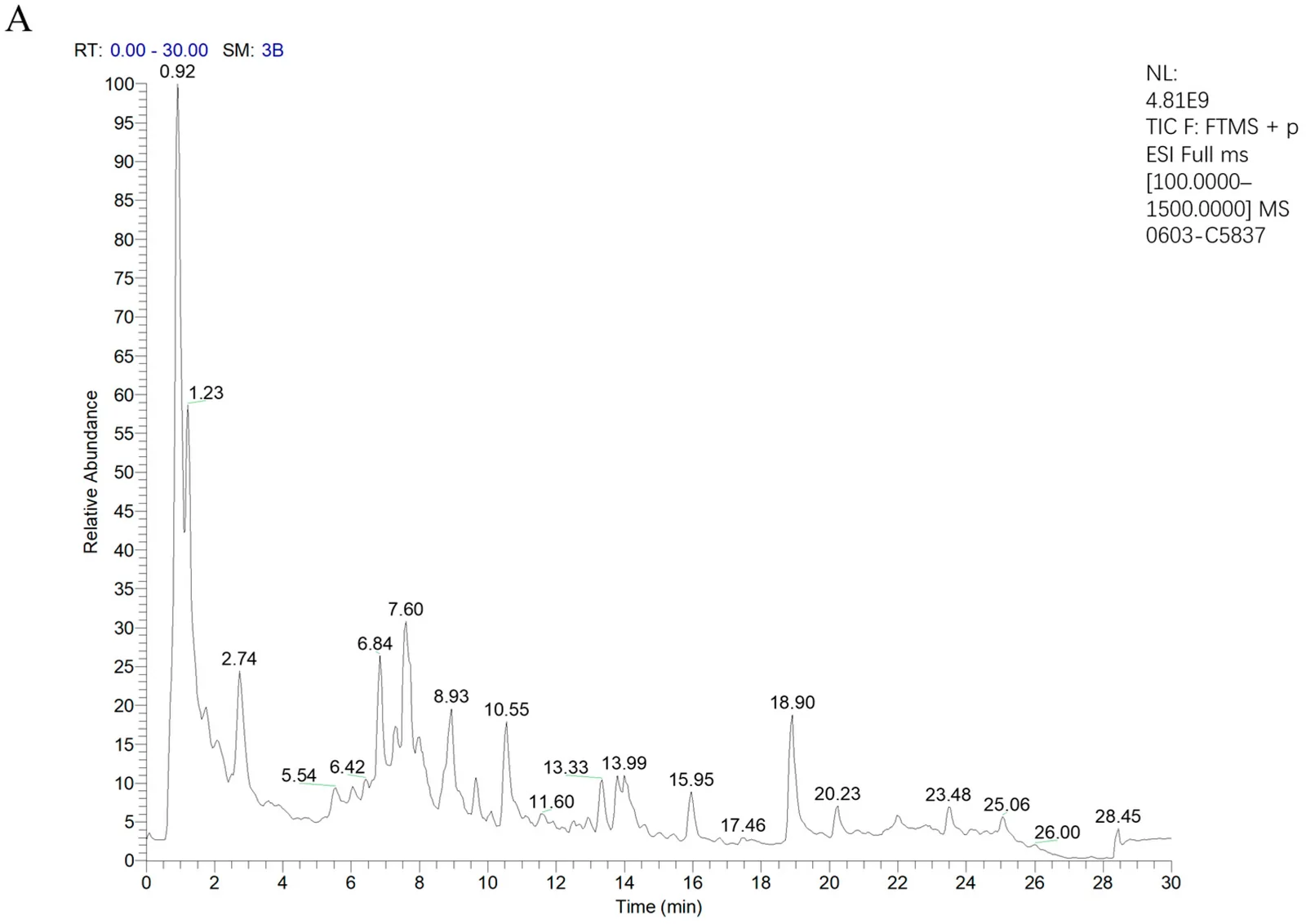

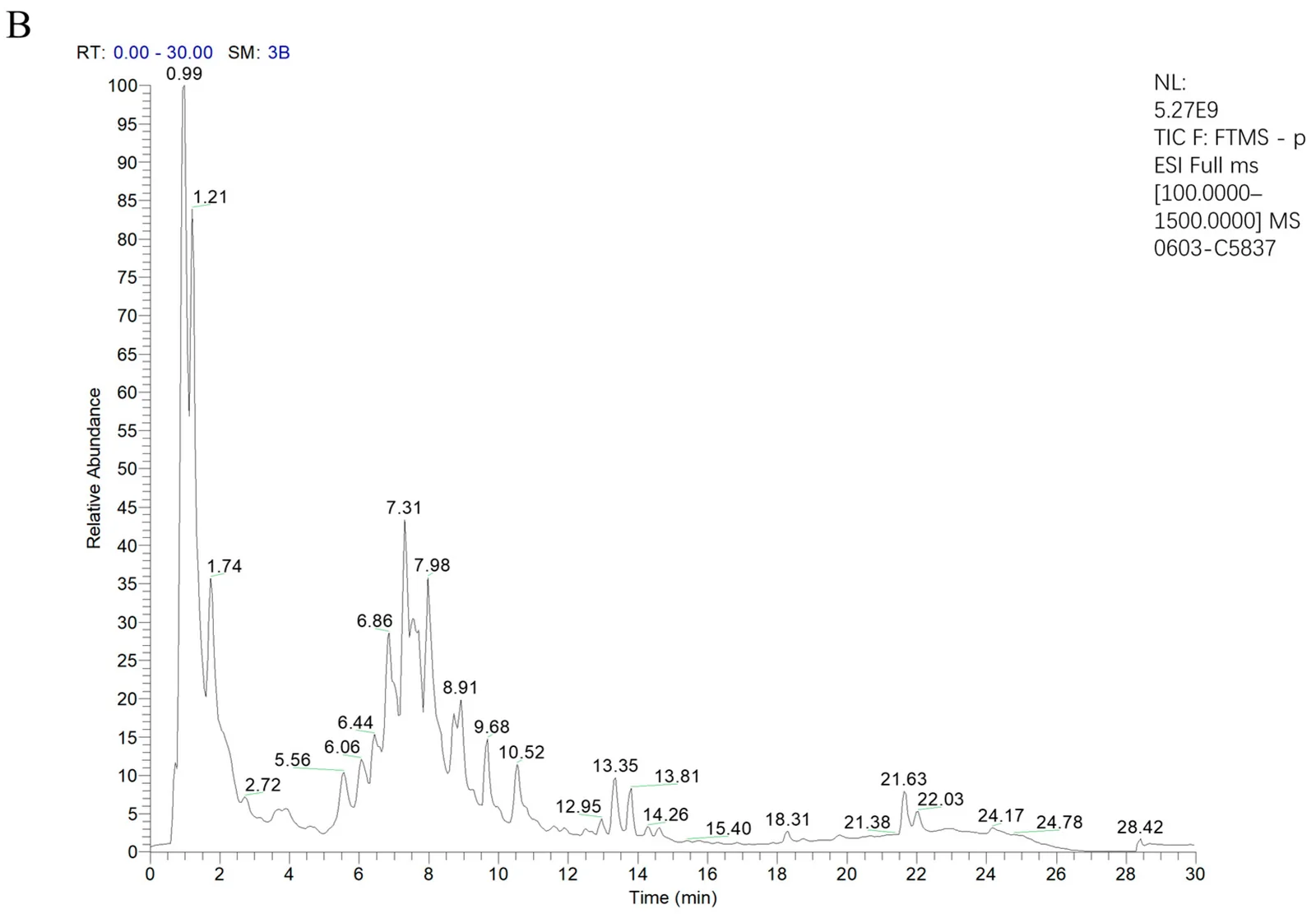
LC-MS total ion chromatograms of Chinese herbal formula F1. (**A**) Total ion chromatogram in positive electrospray ionization mode (ESI+); (**B**) Total ion chromatogram in negative electrospray ionization mode (ESI−).

To analyze the major chemical constituents of Chinese herbal formula F1, HPLC-MS was performed with a detection time of 0–30 min and a mass scan range of *m*/*z* 100–1500. Abundant chromatographic peaks were observed in both positive- and negative-ion modes, and the corresponding total ion chromatograms (TICs) are shown in Figure 1, indicating that F1 contains multiple detectable chemical constituents. After database matching and manual verification of the mass spectrometric data, approximately 1499 annotated compound peaks were obtained. Further screening was performed using a mass error of |ΔMass| < 5 ppm and an mzCloud spectral matching confidence ≥ 80%, resulting in 37 high-confidence candidate compounds.

As shown in Table 6, the five candidate compounds with the highest peak areas were 5-hydroxymethyl-2-furaldehyde, DL-arginine, isoliquiritigenin, 1,6-bis-O-(3,4,5-trihydroxybenzoyl) hexopyranose, and D- (−)-quinic acid. Their maximum peak areas were 8.05 × 10^9^, 4.24 × 10^9^, 3.57 × 10^9^, 2.96 × 10^9^, and 5.76 × 10^8^, respectively. These compounds belonged to several chemical classes, including furan derivatives, amino acids, flavonoids, galloylated polyphenols, and organic acids. Their retention times, observed *m*/*z* values, adduct types, mass errors, mzCloud spectral matching confidence scores, and major MS/MS fragment ions are summarized in Table 6.

It should be noted that LC-MS peak area may be influenced by ionization efficiency, detector response, and other analytical factors and therefore cannot be directly interpreted as absolute compound content or biological importance. Accordingly, the peak areas reported in Table 6 should be regarded as semi-quantitative MS responses only, and absolute quantification using authentic analytical standards was not performed in this study. In addition, the relatively high MS response of 5-hydroxymethyl-2-furaldehyde may reflect either its presence in the herbal materials or its formation during aqueous extraction and heating.

Overall, HPLC-MS analysis revealed a chemically diverse profile of F1, including furan derivatives, amino acids, flavonoids, polyphenolic compounds, and organic acids. These findings primarily characterize the chemical composition of F1 and do not, by themselves, identify the principal antibacterial constituents. Further validation using authentic standards and purified compounds is therefore required.

### 3.5. Effect of Compound Herbal Formula F1 on the Growth Curve of K99-2

**Figure 2:**
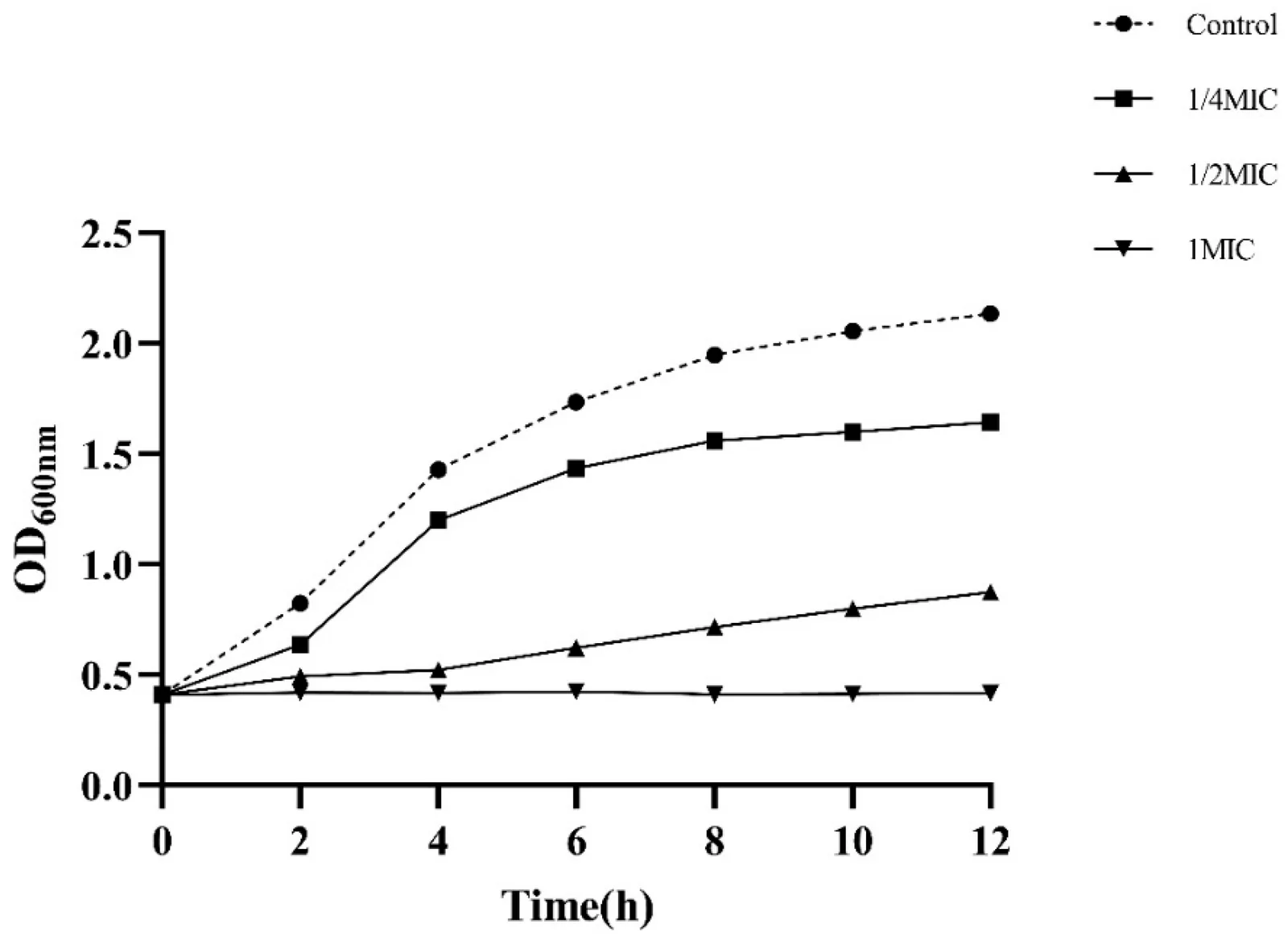
Growth curve of *E. coli* K99-2 treated with Chinese herbal compound F1.

The effect of different concentrations of compound herbal formula F1 on K99-2 growth was evaluated by measuring OD_600nm_ values. The results are shown in Figure 2. The untreated control group exhibited a typical bacterial growth pattern, with rapid increases in OD_600nm_ values during the logarithmic growth phase from 0 to 8 h. After 8 h, the bacteria entered the stationary phase, and the OD_600nm_ values increased only slightly.

The growth trend of the 1/4 × MIC treatment group was similar to that of the control group; however, the OD_600nm_ values at all detection time points were lower than those of the control group, indicating that F1 at this concentration partially inhibited K99-2 growth. The 1/2 × MIC treatment group showed a markedly reduced growth rate, delayed logarithmic phase, and decreased final OD_600nm_ value. In contrast, the OD_600nm_ values of the 1 × MIC treatment group showed no obvious increase during the 12 h monitoring period and remained near the initial level, suggesting marked suppression of K99-2 growth under the tested conditions.

Taken together, compound herbal formula F1 exhibited growth-inhibitory activity against K99-2, and greater growth inhibition was observed at higher F1 concentrations in this experiment. Because bacterial growth was evaluated solely by OD600 in the present experiment, the current data cannot distinguish between bactericidal activity and bacteriostatic inhibition. Viable colony counting (CFU/mL) will be required in future studies to further clarify the antibacterial effect of F1.

### 3.6. Effect of Compound Herbal Formula F1 on Membrane Potential of K99-2 Cells

**Figure 3:**
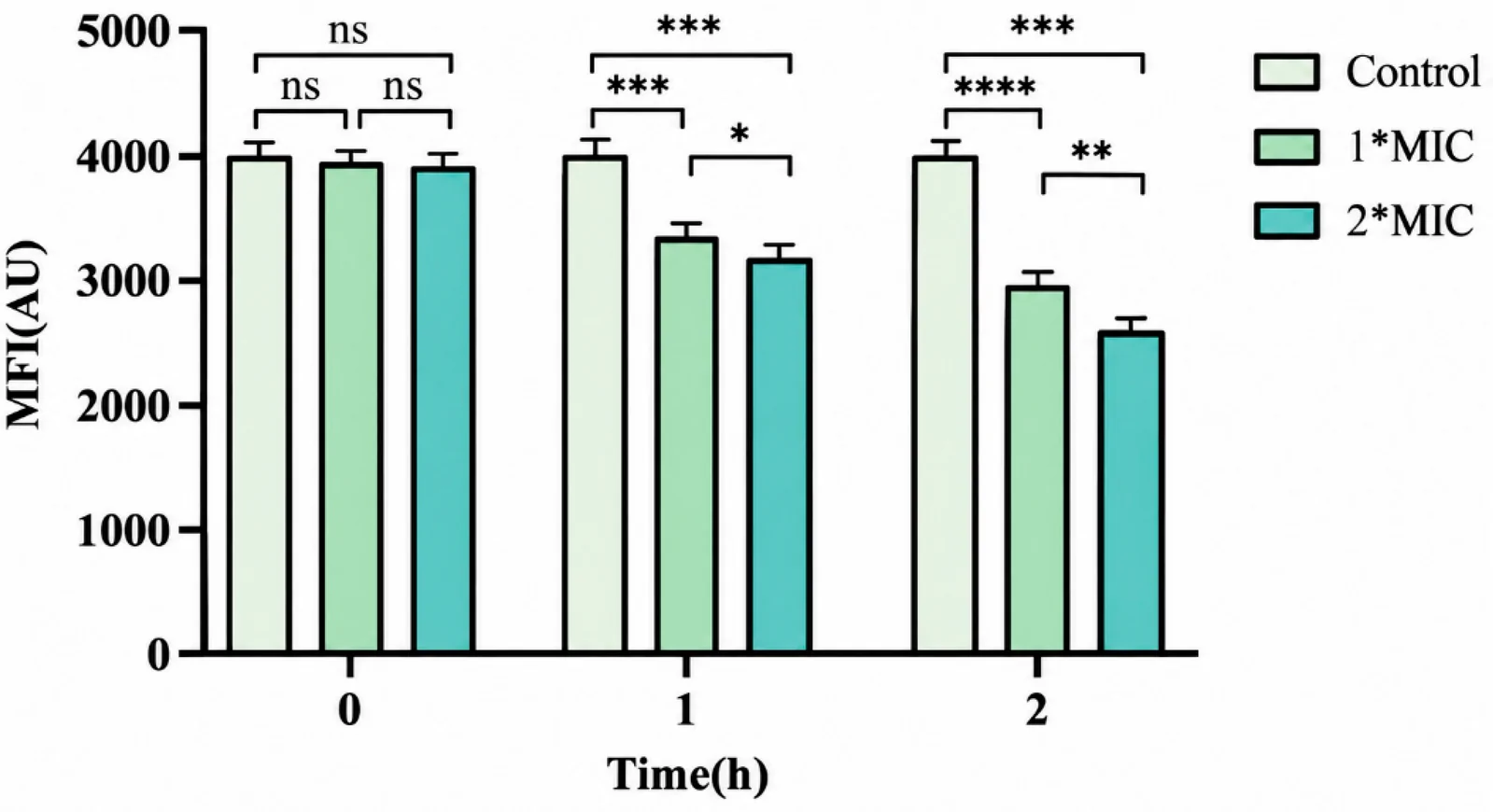
Effect of Chinese herbal compound F1 on membrane potential (MP) of *E. coli* K99-2. Data are presented as mean ± SD (n = 3). Statistical significance was determined using two-way repeated measures ANOVA with Geisser–Greenhouse correction, followed by Sidak’s multiple comparisons test. ns, not significant; * *p* < 0.05, ** *p* < 0.01, *** *p* < 0.001, and **** *p* < 0.0001.

Rhodamine 123 fluorescence staining was used to evaluate the effect of different concentrations of compound herbal formula F1 (1 × MIC and 2 × MIC) on the membrane potential of K99-2 cells. The mean fluorescence intensity (MFI) was used as the indicator, and changes were detected after treatment for 0, 1, and 2 h. The results are shown in Figure 3.

At the initial stage of treatment (0 h), no significant difference in MFI was observed between the F1-treated groups and the blank control group, indicating that F1 did not cause obvious changes in the membrane potential of K99-2 cells at this time point. After treatment for 1 h and 2 h, the MFI values of both the 1 × MIC and 2 × MIC groups were significantly lower than those of the control group. The effect of F1 on membrane potential showed clear concentration- and time-dependent characteristics.

Overall, compound herbal formula F1 induced membrane depolarization in K99-2 cells, resulting in decreased membrane potential. This effect was further enhanced with increasing drug concentration and prolonged treatment time.

### 3.7. Effect of Compound Herbal Formula F1 on AKP Content in K99-2

**Figure 4:**
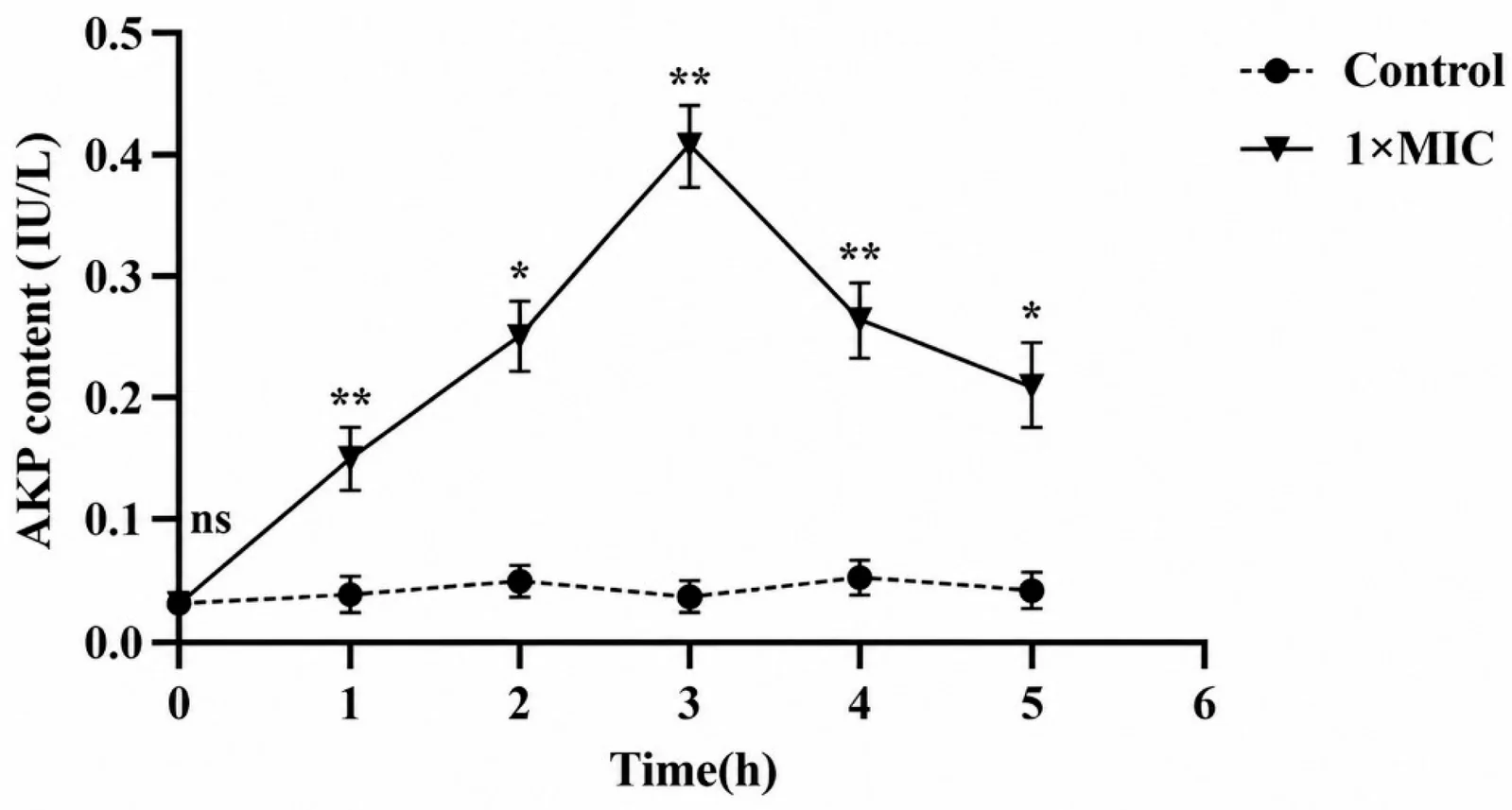
Effect of compound herbal formula F1 on extracellular AKP activity of *E. coli* K99-2. Data are presented as mean ± SD. Statistical significance was determined using two-way repeated measures ANOVA with Geisser–Greenhouse correction, followed by Sidak’s multiple comparisons test. ns, not significant; * *p* < 0.05 and ** *p* < 0.01.

The effect of compound F1 treatment on cell wall permeability of K99-2 was evaluated by measuring extracellular alkaline phosphatase (AKP) activity in bacterial culture supernatants, and the results are shown in Figure 4.

In the untreated control group, extracellular AKP activity remained at a low level with slight fluctuations during 0–5 h, indicating relatively limited AKP leakage under untreated conditions. In contrast, treatment with compound F1 at 1 × MIC markedly increased extracellular AKP activity, with an initial increase followed by a subsequent decline.

The AKP activity in the F1-treated group gradually increased and reached a peak at 3 h (approximately 0.41 IU/L), followed by a gradual decrease. Compared with the control group, F1 treatment resulted in significantly higher AKP activity from 1 to 5 h, suggesting that compound F1 disrupted the cell wall integrity of K99-2, increased cell wall permeability, and promoted AKP release.

After reaching the maximum level at 3 h, AKP activity gradually declined. The present data do not allow the mechanism underlying this decline to be determined.

Overall, compound F1 increased cell wall permeability and promoted AKP leakage in K99-2, suggesting that disruption of cell wall integrity may contribute to the antibacterial activity of compound F1.

### 3.8. Effect of Compound Herbal Formula F1 on Intracellular Protein Content of K99-2

**Figure 5:**
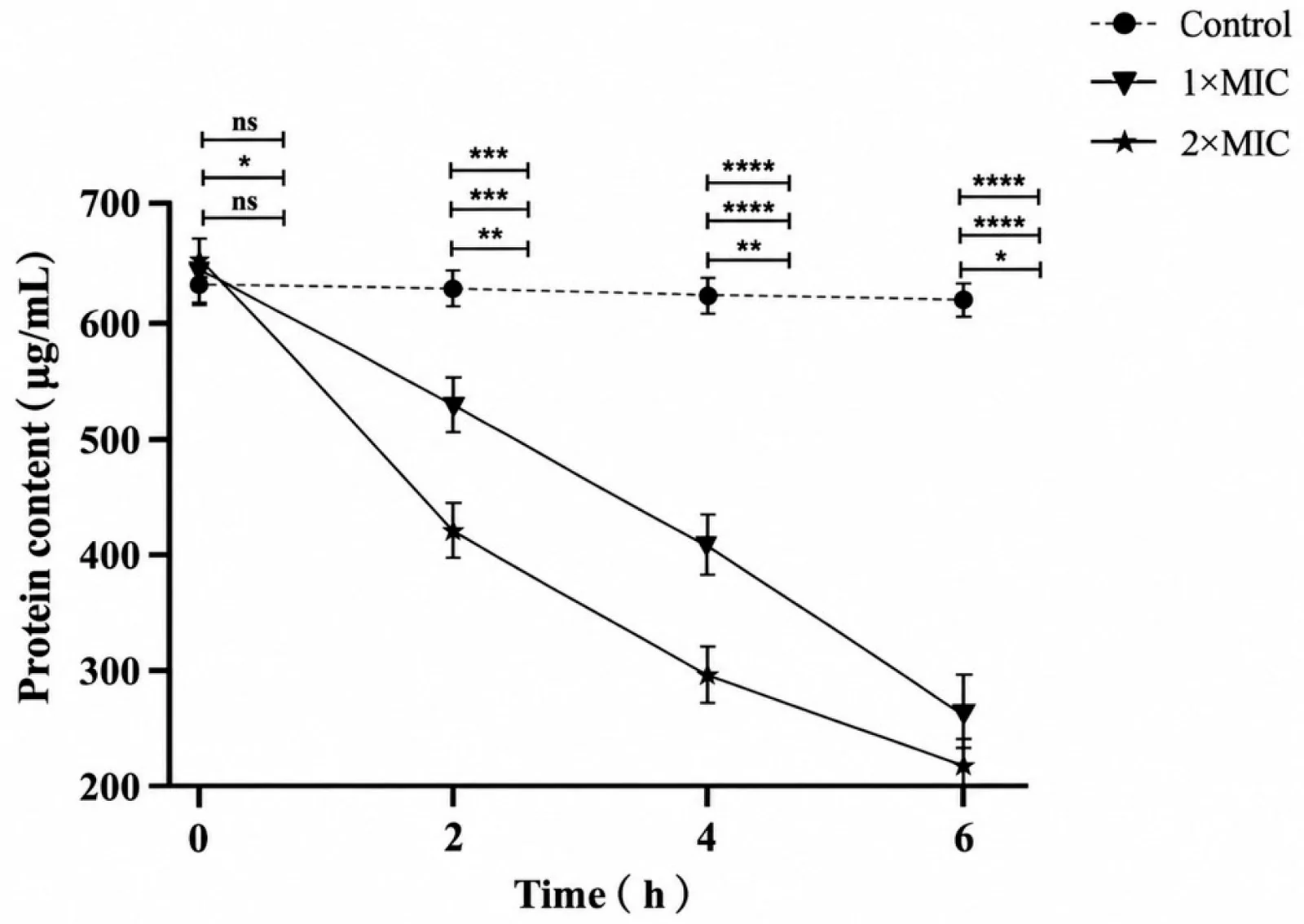
Effect of Chinese herbal compound F1 on intracellular protein content of *E. coli* K99-2. Data are presented as mean ± SD. Statistical significance was determined using two-way repeated measures ANOVA with Geisser–Greenhouse correction, followed by Sidak’s multiple comparisons test. ns, not significant; * *p* < 0.05, ** *p* < 0.01, *** *p* < 0.001, and **** *p* < 0.0001.

Intracellular protein levels were measured to evaluate the effects of different concentrations of F1 (1 × MIC and 2 × MIC) on intracellular protein content in K99-2. The results are shown in Figure 5.

In the blank control group, intracellular protein content remained relatively stable within 0–6 h, indicating relatively stable intracellular protein levels under untreated conditions. After F1 treatment, intracellular protein levels in both the 1 × MIC and 2 × MIC groups continuously decreased with prolonged exposure, showing reductions of 54.91% and 67.46%, respectively. A greater reduction was observed in the 2 × MIC group than in the 1 × MIC group.

Taken together, F1 treatment reduced the intracellular protein content of K99-2, with a greater reduction observed at the higher concentration. These findings suggest that F1 may affect bacterial membrane integrity or intracellular physiological processes. However, because extracellular protein in the culture supernatant was not quantified, the present results do not directly demonstrate intracellular protein leakage.

### 3.9. Effect of Compound Herbal Formula F1 on Intracellular ATP Levels in K99-2

**Figure 6:**
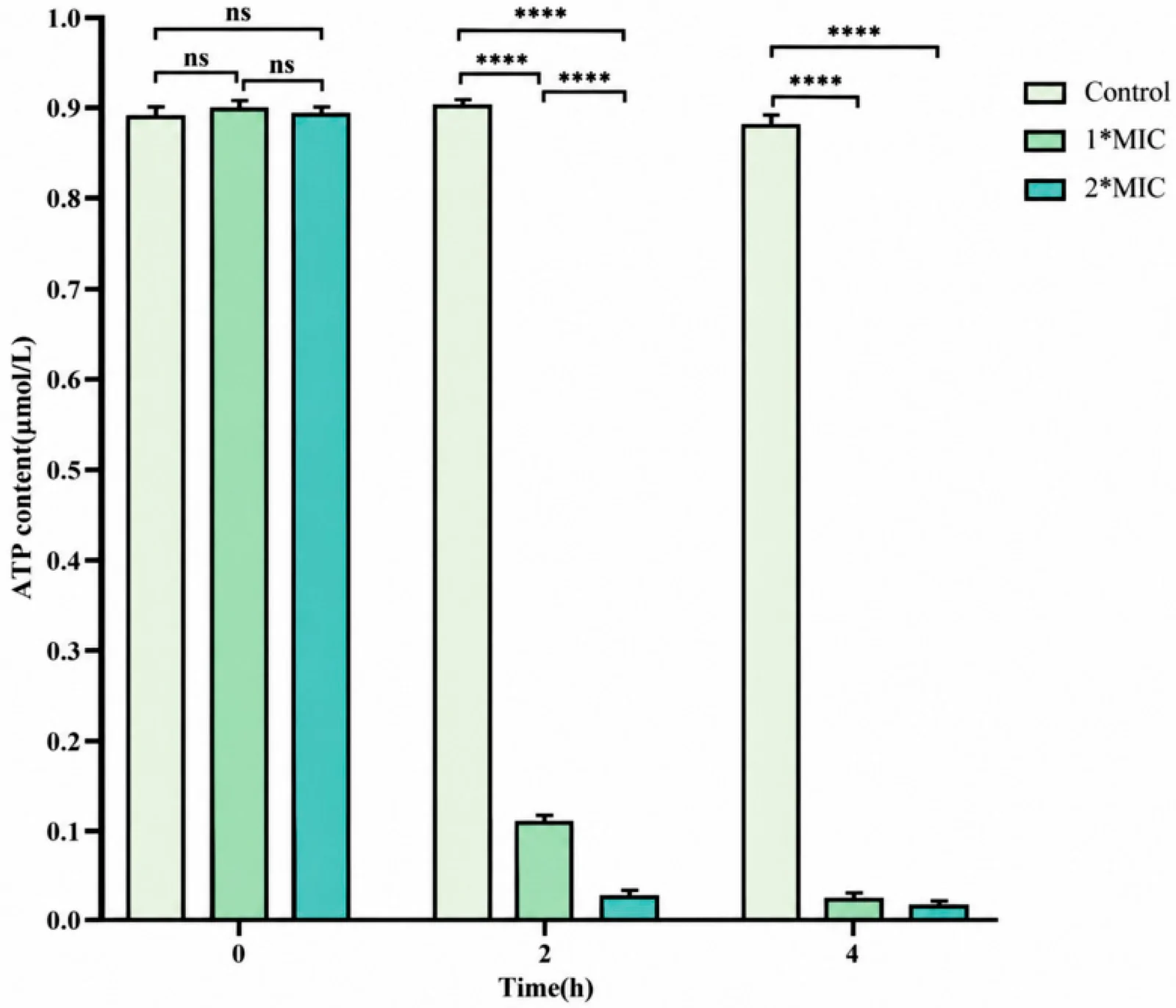
Effect of compound formula F1 on intracellular ATP content of *E. coli* K99-2. Data are presented as mean ± SD (n = 3). Statistical significance was determined using two-way repeated measures ANOVA with Geisser–Greenhouse correction, followed by Sidak’s multiple comparisons test. ns, not significant; * *p* < 0.05, ** *p* < 0.01, *** *p* < 0.001, and **** *p* < 0.0001.

Intracellular ATP levels were measured to investigate the effects of different concentrations of F1 (1 × MIC and 2 × MIC) on intracellular ATP content in K99-2 after treatment for 0, 2, and 4 h. The results are shown in Figure 6.

At 0 h after treatment, intracellular ATP levels in the F1-treated groups showed no significant difference compared with the untreated control group, indicating that no detectable change in intracellular ATP occurred immediately after F1 treatment. After treatment for 2 h and 4 h, intracellular ATP levels in both the 1 × MIC and 2 × MIC groups were significantly decreased and were lower than those in the control group (*p* < 0.0001). Moreover, greater reductions in ATP levels were observed with increasing F1 concentration and prolonged treatment time.

These results suggest that F1 treatment is associated with reduced intracellular ATP levels and may affect bacterial energy metabolism. However, because ATP levels were not normalized to cellular biomass or viable cell counts, the observed decrease may also partly reflect a reduction in the number of intact bacterial cells.

### 3.10. Effect of Compound Herbal Formula F1 on Nucleic Acids of K99-2

**Figure 7:**
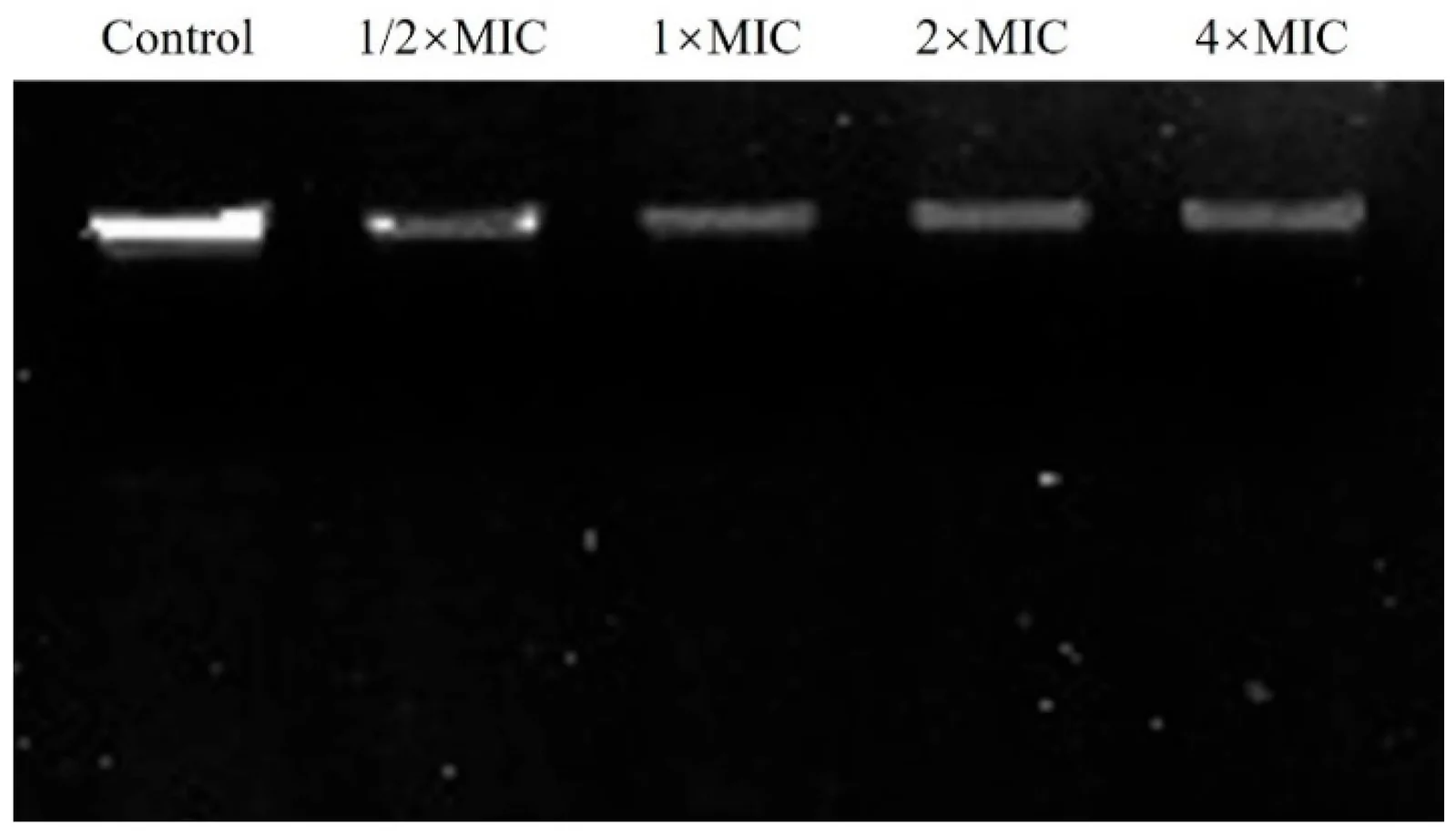
Gel electrophoresis analysis of *E. coli* K99-2 genomic DNA after treatment with Compound Formula F1.

A modified gel retardation assay was performed to evaluate the effect of different concentrations of F1 on genomic DNA of K99-2. The results are shown in Figure 7.

No obvious migration retardation, band delay, or tailing was observed in the DNA electrophoresis bands of any F1 treatment group, indicating that F1 did not form stable complexes with K99-2 genomic DNA that could be maintained during electrophoresis.

### 3.11. Effect of Compound Herbal Formula F1 on the Morphology of K99-2

**Figure 8:**
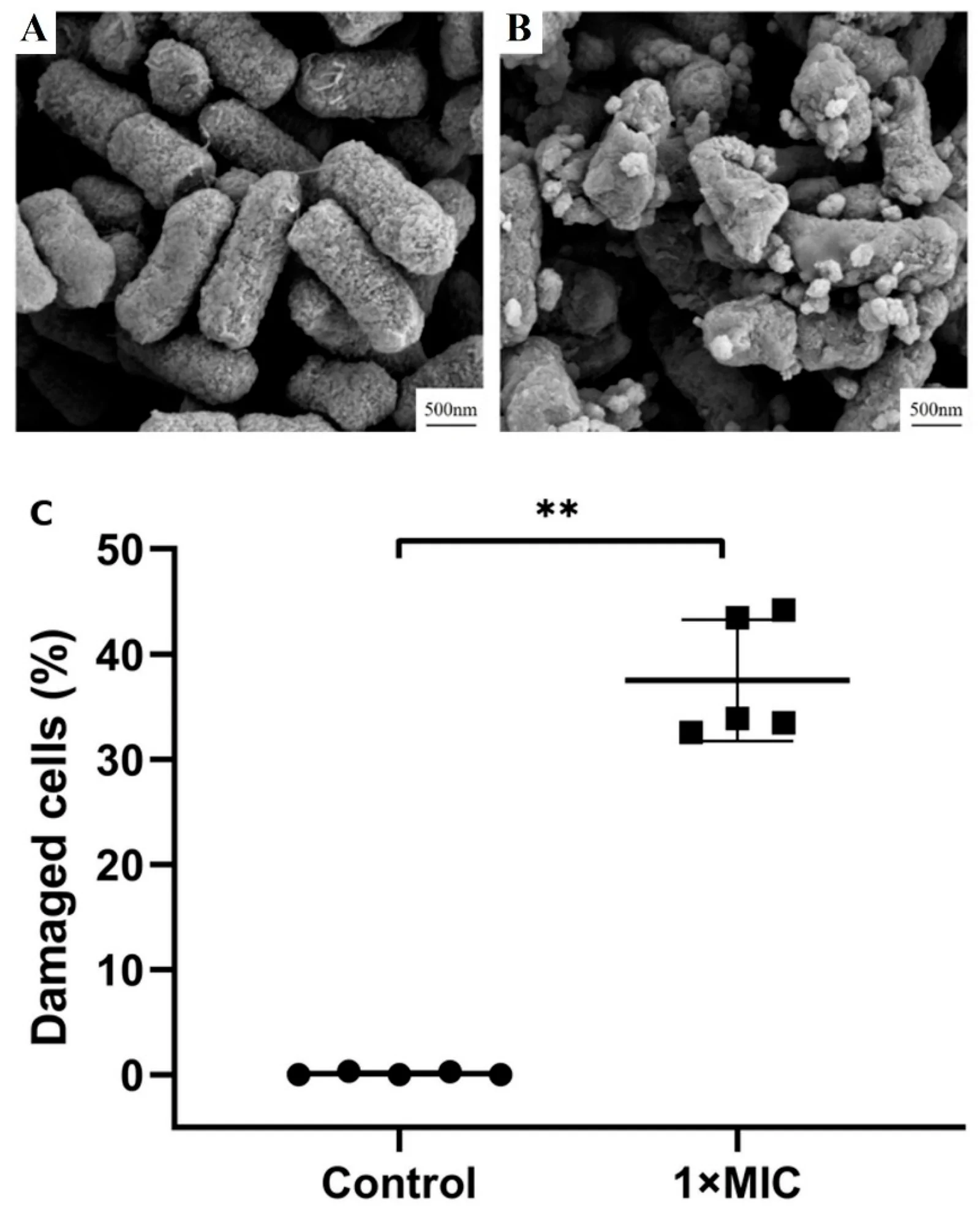
SEM observation and quantitative analysis of K99-2 morphology after F1 treatment. (**A**) Untreated control; (**B**) K99-2 treated with F1 at 1 × MIC for 4 h. Representative SEM images were obtained at 20,000× magnification. (**C**) Percentage of morphologically damaged cells quantified from five independent fields of view per group at 5000× magnification. Data are presented as mean ± SD. Statistical significance was analyzed using the Mann–Whitney test. ** *p* < 0.01.

Scanning electron microscopy (SEM) was used to observe morphological changes in K99-2 cells before and after treatment with compound herbal formula F1 and to evaluate its effects on bacterial ultrastructure. The results are shown in Figure 8.

In the untreated control group, K99-2 cells exhibited typical short rod-shaped morphology, with relatively uniform size and regular arrangement. The cell surface was smooth and intact, and the cell wall and membrane structures remained well preserved. No obvious shrinkage, rupture, or morphological abnormalities were observed (Figure 8A). In contrast, treatment with F1 at 1 × MIC caused obvious ultrastructural alterations in K99-2 cells. The treated cells showed varying degrees of shrinkage, deformation, and irregular collapse. Some cells exhibited rough surfaces, obvious depressions, and structural damage. The integrity of bacterial cells was disrupted, and some cells showed fragmentation and breakage (Figure 8B).

Quantitative analysis of five independent fields of view showed that the proportion of morphologically damaged cells was significantly higher in the F1-treated group than in the untreated control group (*p* = 0.0079; Figure 8C).

These results indicate that compound herbal formula F1 induced marked morphological damage in K99-2 cells and affected bacterial structural integrity.

### 3.12. 28-Day Oral Toxicity Study

In this study, Kunming mice were orally administered different concentrations of compound herbal formula F1 by gavage. The maximum concentration was set at 1000 mg/mL, and treatment was continued for 28 days to evaluate its long-term oral safety. During the experimental period, no mortality was observed in any concentration group. No obvious abnormalities were detected in the general condition, spontaneous activity, food and water intake, or fecal characteristics of mice. According to the predefined clinical observation criteria, all evaluated clinical parameters showed a score of 0, indicating the absence of abnormal clinical signs during the experimental period (Table 7).

Compared with the untreated control group, mice treated with the high- concentration F1 showed no obvious changes in fur appearance or response to external stimuli. No typical toxic symptoms, such as curling or convulsions, were observed. Throughout the experiment, no toxicity-related clinical signs, including increased ocular or nasal secretions, abnormal skin or mucosal conditions, inhibited body weight gain, or body weight loss, were observed in any group (Table 7).

These results indicate that continuous oral administration of F1 at 1000 mg/mL for 28 days did not cause obvious toxic effects in Kunming mice under the present experimental conditions, demonstrating good oral safety.

### 3.13. Analysis of Blood Biochemical Parameters

**Figure 9:**
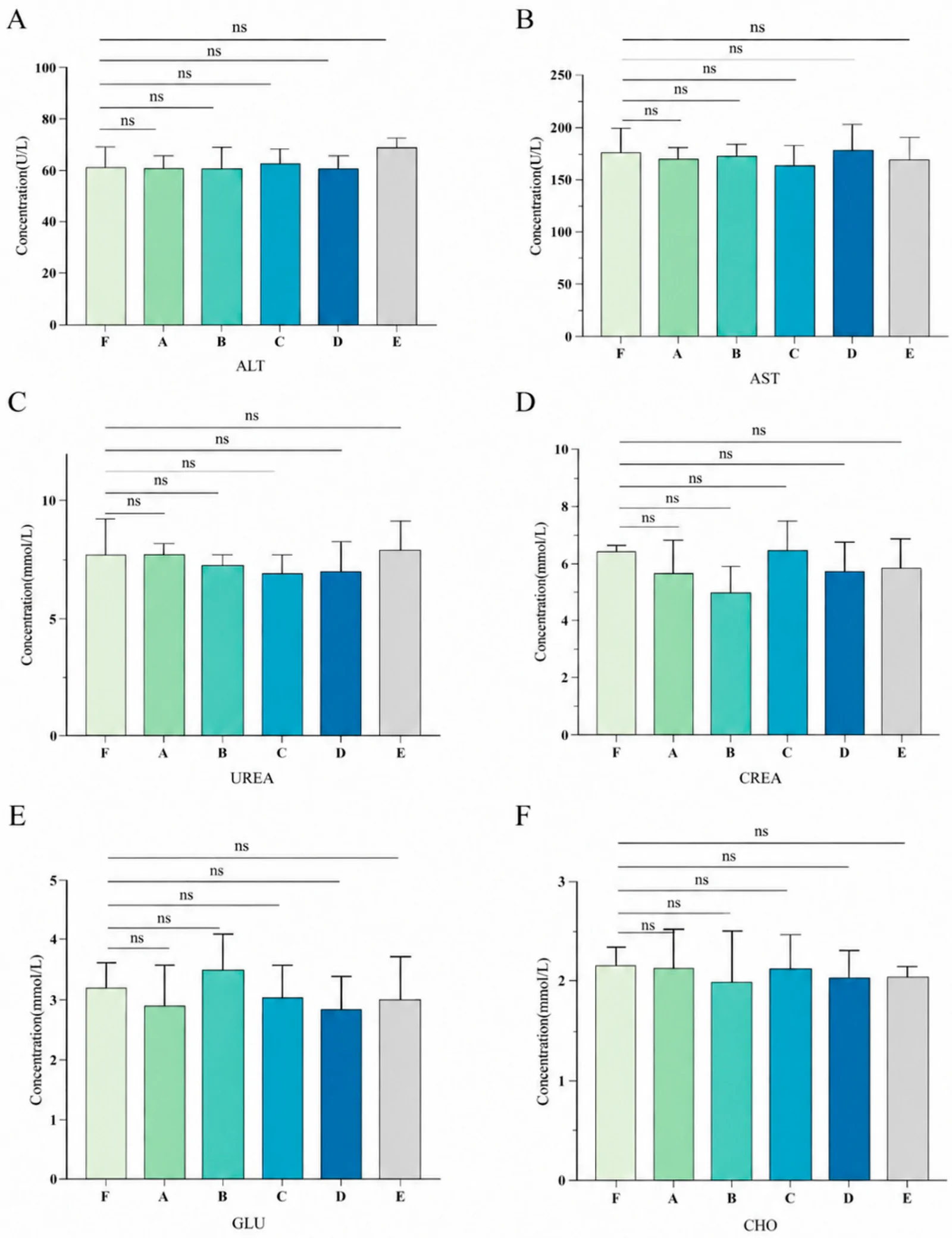
Serum biochemical parameters of mice in different experimental groups. (**A**) Comparison of ALT levels among different groups of mice; (**B**) Comparison of AST levels among different groups of mice; (**C**) Comparison of UREA levels among different groups of mice; (**D**) Comparison of CREA levels among different groups of mice; (**E**) Comparison of GLU levels among different groups of mice; (**F**) Comparison of CHO levels among different groups of mice. Data are presented as mean ± SD (n = 5). Statistical significance was determined using one-way ANOVA followed by Tukey’s multiple comparisons test. ns, not significant; * *p* < 0.05, ** *p* < 0.01, *** *p* < 0.001, and **** *p* < 0.0001.

The results of hematological parameters and serum biochemical indicators are shown in Table 8 and Figure 9, respectively. After 28 days of continuous gavage administration, all hematological parameters in the F1-treated groups (groups A–E) remained within normal physiological ranges. Compared with the untreated control group (group F), no significant differences were observed in white blood cell count (WBC), red blood cell count (RBC), hemoglobin concentration (HGB), platelet count (PLT), or leukocyte differential counts (*p* > 0.05).

Serum biochemical analysis further showed that liver and kidney function-related indicators, including alanine aminotransferase (ALT), aspartate aminotransferase (AST), total bilirubin (TBIL), creatinine (CREA), blood urea nitrogen (BUN), total protein (TP), albumin (ALB), and glucose (GLU), showed no abnormal changes in mice treated with different concentrations of F1. No significant differences were observed compared with the control group (*p* > 0.05).

These results demonstrate that F1 administration at the tested concentration range (≤1000 mg/mL) for 28 days did not adversely affect the hematopoietic system, coagulation function, or hepatic and renal metabolism, further confirming its favorable oral safety.

### 3.14. Organ Index Measurement

The potential effects of continuous F1 administration on major organs were evaluated by measuring the organ indices of the heart, liver, spleen, lung, and kidney in mice. The results are shown in Table 9.

Compared with the untreated control group (PBS group), no significant differences were observed in the relative organ indices of the heart and liver in F1-treated groups. Although statistically significant differences were observed in some individual organ parameters, these changes were not associated with consistent dose-dependent alterations.

These results indicate that continuous oral administration of F1 for 28 days did not induce obvious abnormal changes in major organs, suggesting good organ safety within the tested dose range.

### 3.15. Histopathological Observation of Ileum Tissue

**Figure 10:**
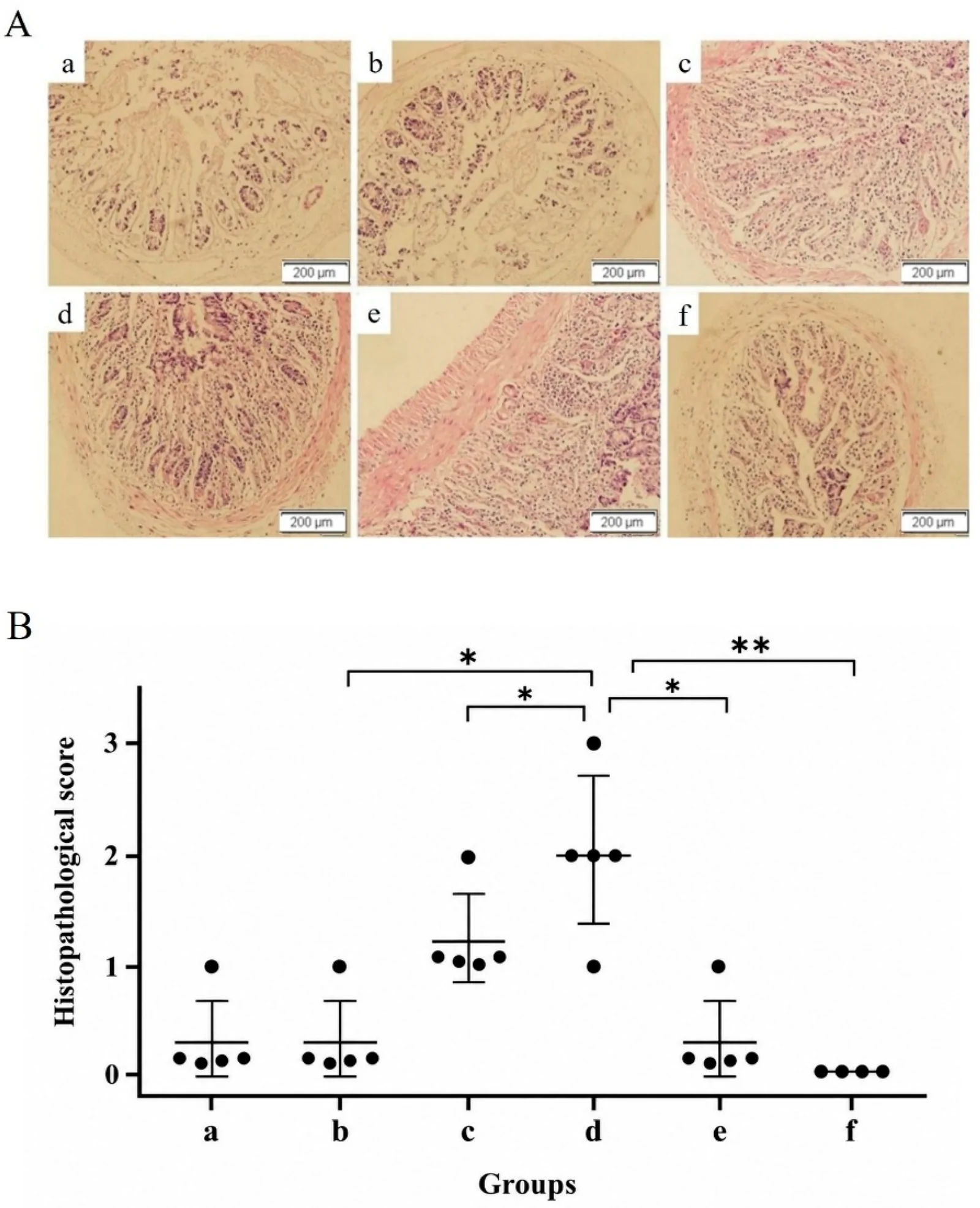
Histopathological examination and semi-quantitative scoring of ileum tissues. (**A**) H&E staining of ileum tissues (200×). (**B**) Semi-quantitative histopathological scores of ileum tissues. Data are presented as mean ± SD. (**a**) Group A; (**b**) Group B; (**c**) Group C; (**d**) Group D; (**e**) Group E; (**f**) Group F. Statistical significance was determined using the Kruskal–Wallis test followed by Dunn’s multiple comparisons test. * *p* < 0.05 and ** *p* < 0.01,

Histopathological changes in mouse ileum tissues are shown in Figure 10. After 28 days of continuous gavage administration, the ileum structures remained generally intact in F1-treated groups (groups A–E) and the untreated control group (group F).

H&E staining showed that the ileal mucosal structures were intact in all groups. The intestinal villi were regularly arranged, and epithelial cells maintained normal morphology. No severe pathological changes, including marked mucosal injury, epithelial necrosis, extensive inflammatory infiltration, or obvious villus atrophy, were observed. To further evaluate histological changes objectively, semi-quantitative histopathological scoring was performed based on mucosal damage, epithelial degeneration, inflammatory cell infiltration, and villus abnormalities. The stained sections were independently evaluated by two researchers who were blinded to the experimental groups. The histopathological scores were 0.20 ± 0.45, 0.20 ± 0.45, 1.20 ± 0.45, 2.00 ± 0.71, 0.20 ± 0.45, and 0.00 ± 0.00 for groups A, B, C, D, E, and F, respectively. The Kruskal–Wallis test showed a significant overall difference among the six groups (H = 21.82, *p* = 0.0006). Dunn’s multiple comparisons test further showed that group D had significantly higher histopathological scores than groups A, B, E, and F (*p* < 0.05), whereas the remaining pairwise comparisons were not statistically significant. Mild increases in histopathological scores were observed in groups C and D, whereas groups A, B, and E showed scores close to the control group.

These results indicate that continuous oral administration of F1 for 28 days did not cause severe histological abnormalities in the mouse ileum within the tested dose range, although mild histopathological changes were observed in some dose groups, particularly group D.

### 3.16. Determination of LD_50_ of K99-2

A mouse infection model was established by intraperitoneal injection of K99-2, and clinical symptoms and mortality were continuously monitored for 14 days to evaluate the systemic pathogenic potential of K99-2. The results showed that 6 h after infection, mice challenged with high bacterial doses (10^8^ CFU/mouse and 10^7^ CFU/mouse) gradually developed obvious infection symptoms, including depression, reduced food intake, eyelid swelling with increased purulent secretion, and decreased activity. At 24 h post-infection, some infected mice developed diarrhea accompanied by abnormal fecal adhesion around the anus (Figure 10). From the first day after infection, mortality occurred successively in all challenged groups.

Based on the mortality data of mice in different challenge groups (Table 10), the median lethal dose (LD_50_) of K99-2 infection in mice was calculated using the modified Karber method. The LD_50_ of K99-2 in Kunming mice following intraperitoneal injection was 6.9 × 10^7^ CFU/mouse.

These results demonstrate that K99-2 exhibits systemic pathogenic potential following intraperitoneal infection and can induce acute infection symptoms and mortality within a certain dose range, providing a reliable infection model for subsequent evaluation of the protective effect of F1 in vivo.

### 3.17. Effect of Compound Herbal Formula F1 on Mortality of K99-2-Infected Mice

**Figure 11:**
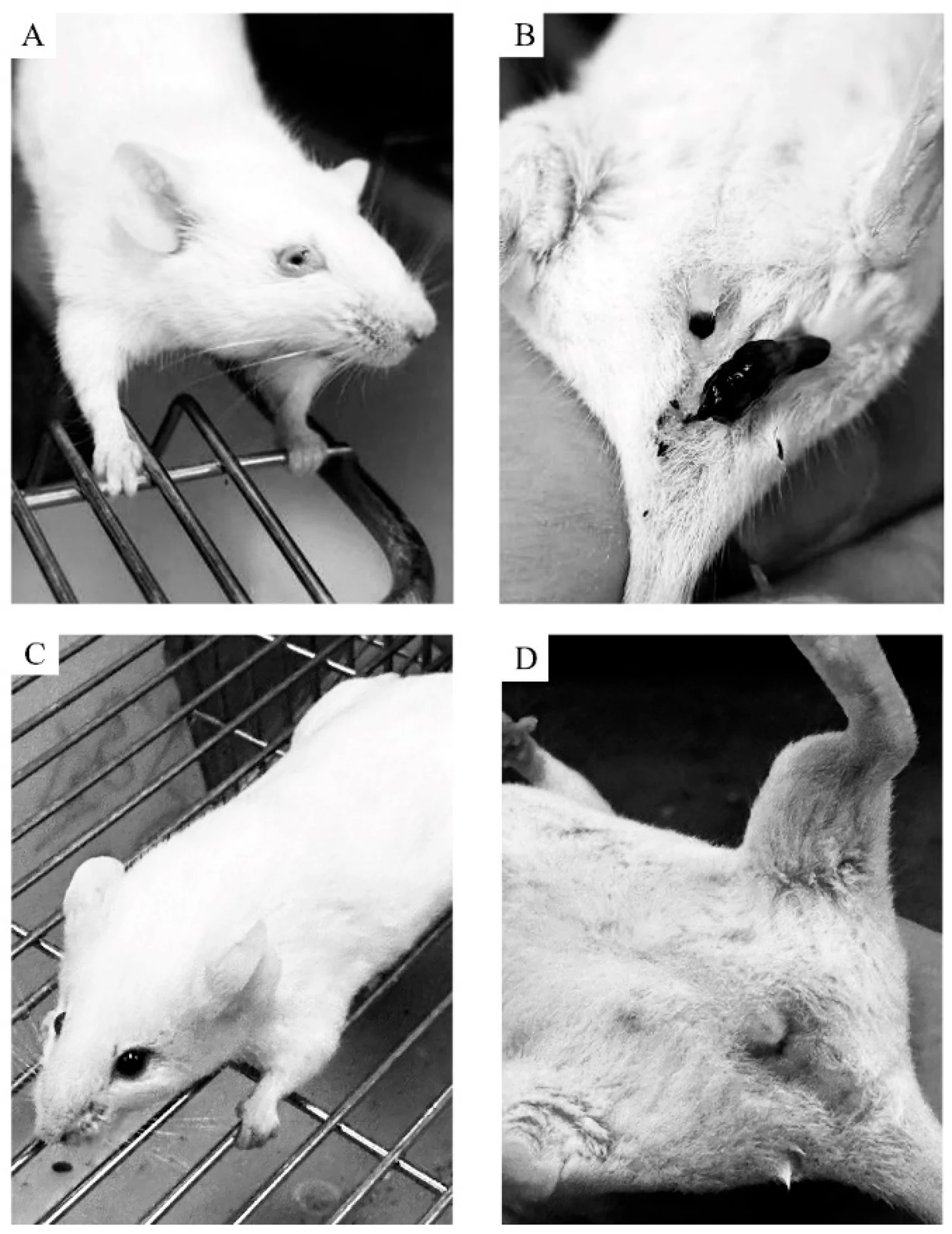
Clinical symptoms of challenged mice. (**A**) Eyelid swelling accompanied by increased purulent discharge; (**B**) Fecal adhesion around the anus with abnormal fecal characteristics; (**C**) Normal eyelid appearance of healthy mice; (**D**) Normal perianal appearance of healthy mice.

The 7-day survival rates of mice are shown in Figure 11. No mortality occurred in the blank control group (group C) during the entire observation period, with a survival rate of 100%. In contrast, the infected group without treatment (group B) showed a high mortality rate, with a 7-day survival rate of only 40%. Compared with the infected group, mice treated with F1 (group A) showed an increased 7-day survival rate of 73%, which was significantly higher than that of group B.

**Figure 12:**
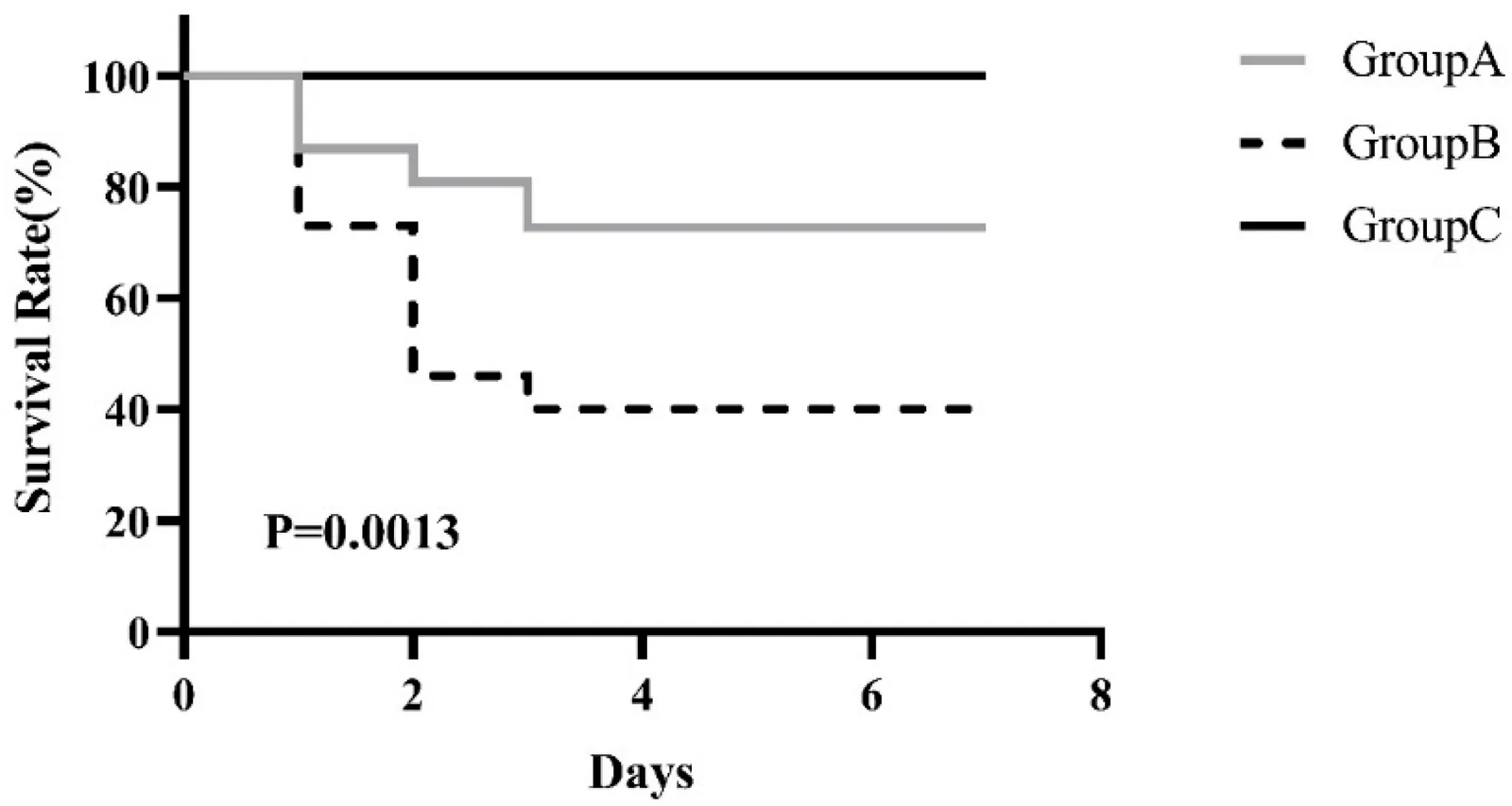
Survival curves of mice in different groups within 7 days. Data are presented as survival percentage. Survival curves were generated using the Kaplan–Meier method and compared using the Log-rank (Mantel–Cox) test. *p* < 0.05 was considered statistically significant.

Statistical analysis showed that F1 treatment significantly improved the survival of K99-2-infected mice and reduced infection-associated mortality (Figure 12). These results indicate that compound herbal formula F1 provides effective in vivo protection against K99-2 infection, alleviates infection-induced lethal damage, and exhibits promising therapeutic potential.

## 4. Discussion

Enterotoxigenic *Escherichia coli* (ETEC) carrying K99 fimbriae is one of the major pathogens responsible for neonatal calf colibacillosis. This disease exhibits high incidence and mortality among newborn calves, severely threatening calf health and causing substantial economic losses to the cattle industry [30,31].

Chinese herbal medicines possess advantages including wide availability, diverse chemical compositions, and multiple biological targets. They have demonstrated potential antibacterial, anti-inflammatory, immunomodulatory, and tissue repair activities. Therefore, screening active compounds or herbal combinations from traditional Chinese medicines against *E. coli* K99 represents an important strategy for developing novel veterinary antimicrobial agents.

In this study, compound herbal formula F1 was obtained through systematic screening and showed inhibitory activity against *E. coli* K99. Further investigations demonstrated that F1 induced membrane depolarization, disrupted bacterial cell wall and membrane integrity, and interfered with bacterial energy metabolism. Meanwhile, F1 exhibited favorable safety profiles and improved the survival of K99-infected mice. These findings indicate that F1 exhibited antibacterial activity against bovine-derived *E. coli* K99-2 and was associated with multiple antibacterial processes, including disruption of bacterial cell-envelope integrity, membrane depolarization, and interference with energy metabolism.

The antibacterial effects of single herbal medicines are usually associated with limited targets and depend mainly on specific active compounds. In contrast, compound herbal formulas contain more complex chemical components and may exert antibacterial effects through the combined actions of multiple constituents. Such interactions can simultaneously affect bacterial structures, energy metabolism, and host inflammatory responses [32,33]. In this study, the antibacterial activities of 70 individual Chinese herbal medicines were initially screened, followed by formula optimization based on traditional compatibility principles. The results showed that compound herbal formula F1 had an inhibition zone diameter of 21 mm, with both MIC and MBC values of 62.5 mg/mL. Although this concentration is relatively high compared with that typically reported for purified antimicrobial agents, F1 is a complex aqueous herbal extract containing multiple constituents, and its mass-based antibacterial activity should therefore be interpreted with caution. Further studies are needed to identify and quantify the major active components and to evaluate the antibacterial activity of standardized extracts or purified compounds. Moreover, the relatively high in vitro inhibitory concentration of F1 should be carefully considered when evaluating its practical veterinary application. The effective concentration observed in vitro may not directly represent the required therapeutic dose in production animals, and further pharmacokinetic studies and dosage optimization are needed to determine its therapeutic feasibility.

Because the antibacterial activity of herbal medicines may be associated with their chemical composition, LC-MS was used in this study to preliminarily characterize the chemical profile of Chinese herbal formula F1 and annotate candidate compounds [34]. The results revealed several classes of candidate compounds, including furan derivatives, amino acids, flavonoids, galloylated polyphenols, and organic acids. Previous studies have reported that polyphenolic compounds may exert antibacterial effects by inhibiting biofilm formation or inducing enzyme inactivation [22], while organic acids can affect bacterial membrane function and alter intracellular proton and anion concentrations, thereby contributing to antibacterial activity [35]. Flavonoids have also been reported to possess anti-inflammatory activity through modulation of the NF-κB and MAPK signaling pathways and downregulation of COX-2, iNOS, and other inflammatory mediators [16]. Among the candidate constituents identified in F1, compounds such as gallic acid and isoliquiritigenin have been reported to exhibit antibacterial activities in previous studies, potentially through effects on bacterial membrane integrity, oxidative stress, or metabolic processes [36]. Similarly, organic acids such as citric acid and quinic acid may influence bacterial physiological homeostasis through alterations in intracellular pH and membrane-associated functions [37]. These potential activities may be related to the membrane depolarization, cellular damage, and ATP reduction observed after F1 treatment [38,39]; however, the direct contribution of individual constituents requires further validation using purified compounds or bioactivity-guided fractionation. These findings suggest that some constituents belonging to the chemical classes detected in F1 may possess antibacterial or other biological activities [40]. However, the detection of these candidate compounds does not directly demonstrate that they are the principal contributors to the antibacterial activity observed in the present study.

It should also be noted that untargeted LC-MS analysis primarily provides putative chemical annotation rather than definitive identification or direct evidence of biological activity. Peak areas are influenced by ionization efficiency, detector response, and other analytical factors and therefore cannot be interpreted as absolute concentrations or relative biological importance. Further studies using authentic standards, targeted quantitative analysis, isolation of individual compounds or active fractions, and bioactivity-guided evaluation are required to identify the major active constituents of F1 and clarify their contributions to its antibacterial effects.

5-Hydroxymethyl-2-furaldehyde (5-HMF) showed a relatively high MS response in F1. However, 5-HMF may occur naturally in plant-derived materials or may also be generated through sugar degradation during thermal processing. Given that F1 was prepared by repeated aqueous heating followed by autoclaving, part of the detected 5-HMF may have been formed during extraction or sterilization rather than being exclusively derived from the original herbal materials. Therefore, its relatively high MS response should not be directly interpreted as evidence that 5-HMF is a major natural bioactive constituent of F1. Further comparison of unheated, heated, and sterilized samples will be necessary to clarify its origin. Moreover, the contribution of 5-HMF to the antibacterial activity and potential safety profile of F1 remains unclear and requires further investigation using purified 5-HMF and comparative biological evaluation.

The bacterial cell wall is an essential structure responsible for maintaining cell morphology, regulating substance transport, and maintaining intracellular homeostasis. It also serves as an important target for various antimicrobial agents and natural compounds [21,35]. In this study, F1 treatment significantly increased extracellular AKP levels in K99-2, suggesting altered cell-envelope permeability and possible leakage of intracellular or periplasmic components. Furthermore, SEM analysis revealed obvious morphological alterations in F1-treated K99-2 cells, including shrinkage, deformation, surface roughness, local depression, and cell rupture. These observations indicate that F1 treatment was associated with damage to bacterial surface structures; however, the present results are insufficient to define the precise molecular process by which the cell wall was affected.

The bacterial cell membrane plays a critical role in maintaining substance transport, energy conversion, and cellular homeostasis, and its structural integrity is essential for bacterial survival [21,37,41]. In this study, membrane potential analysis showed that F1 significantly reduced the membrane potential of K99-2 cells in a concentration- and time-dependent manner, suggesting membrane depolarization [42]. Meanwhile, intracellular protein levels were markedly reduced after F1 treatment, with decreases of 54.91% and 67.46% in the 1 × MIC and 2 × MIC groups, respectively. These changes are consistent with impaired membrane integrity or altered membrane-associated physiological function [43], although they do not establish the specific molecular events responsible for membrane damage.

Collectively, the present findings support an association between F1 treatment and cell-envelope damage in K99-2, as reflected by increased extracellular AKP levels, morphological alterations, reduced membrane potential, and decreased intracellular protein content. These changes may contribute to the antibacterial activity of F1 by compromising bacterial structural integrity and cellular homeostasis [44]. Jiang et al. [35] reported that plant extracts could induce structural alterations in the cell envelope of *E. coli*, which is generally consistent with the observations of the present study. Nevertheless, the specific molecular targets and pathways involved in F1-induced cell-envelope damage remain to be further investigated.

To further investigate whether F1 exerted antibacterial effects through direct interaction with bacterial nucleic acids, a gel retardation assay was performed to analyze the interaction between F1 and genomic DNA of K99-2. The results showed that no obvious migration retardation, band delay, tailing, or diffusion was observed in DNA electrophoresis bands after treatment with different concentrations of F1. These findings indicate that F1 did not form stable complexes with K99-2 genomic DNA under the experimental conditions.

These results suggest that the antibacterial activity of F1 was not primarily mediated through direct binding to or destruction of bacterial genetic material. Unlike the study by Deng et al. [45], which reported that chokeberry anthocyanins exerted antibacterial effects through direct interaction with bacterial DNA, F1 may mainly inhibit bacterial growth by disrupting cellular structural stability and interfering with essential metabolic processes. Therefore, F1 did not show an obvious direct DNA-binding pattern, and its antibacterial effects may be more closely associated with alterations in other cellular structures or physiological processes.

Adenosine triphosphate (ATP) is an essential energy molecule that participates in various cellular processes, including bacterial growth, reproduction, substance transport, protein synthesis, and maintenance of cellular homeostasis. It is indispensable for the survival of *E. coli* [46]. Inhibition of ATP production or disruption of energy metabolism can effectively weaken bacterial viability and induce antibacterial effects. In this study, F1 significantly reduced intracellular ATP levels in K99-2, and this inhibitory effect was enhanced with increasing drug concentration and prolonged treatment time. These results indicate that interference with bacterial energy metabolism may contribute to the antibacterial activity of F1. Xiumei Yang et al. [47] reported that luteolin treatment significantly decreased intracellular ATP production in *E. coli*, and this inhibitory effect increased with increasing luteolin concentration. Therefore, our findings are consistent with previous studies, suggesting that disruption of energy metabolism may represent one of the mechanisms associated with the antibacterial activity of F1.

Safety evaluation is an essential basis for assessing the application potential and clinical translation value of candidate veterinary drugs [48,49]. In this study, the oral safety of F1 was systematically evaluated through a 28-day oral toxicity study. The results showed that continuous administration of F1 at concentrations ranging from 62.5 to 1000 mg/mL for 28 days caused no mortality, and no abnormal changes were observed in general behavior, spontaneous activity, food and water intake, or fecal characteristics of mice. Further analyses showed that hematological parameters, including white blood cells, red blood cells, hemoglobin, platelets, and leukocyte differential counts, remained within normal physiological ranges in all F1-treated groups. Meanwhile, liver and kidney function-related biochemical indicators, including alanine aminotransferase, aspartate aminotransferase, total bilirubin, creatinine, blood urea nitrogen, total protein, albumin, and glucose, showed no significant differences compared with the control group (*p* > 0.05). In addition, organ index analysis and ileal histopathological examination revealed no obvious abnormalities in major organs or intestinal tissues of treated mice.

These results demonstrate that continuous oral administration of F1 for 28 days within the tested dose range did not adversely affect the overall health status, hematological function, hepatic and renal metabolism, or intestinal tissue structure of mice, indicating favorable preliminary oral safety. Previous studies have indicated that drug toxicity evaluation should consider multiple parameters, including clinical manifestations, physiological indicators, biochemical parameters, and histopathological changes, rather than relying on a single indicator alone [50,51]. Therefore, this study comprehensively evaluated the preliminary safety of F1 using multiple approaches, providing experimental evidence for its further development. However, due to the complex composition and broad biological effects of herbal formulas, further systematic toxicological studies are required to evaluate their long-term safety, species-specific effects, and safety under disease conditions.

The protective efficacy of candidate anti-infective agents in animal models is an important indicator for evaluating their in vivo effectiveness [52,53]. In this study, a K99-2 infection model was first established in mice. Clinical symptoms and mortality were monitored after intraperitoneal challenge, and the median lethal dose (LD_50_) of K99-2 was calculated using the modified Karber method. However, it should be noted that the intraperitoneal infection model used in this study primarily reflects acute systemic infection characteristics and does not fully reproduce the natural pathogenesis of ETEC K99-associated calf diarrhea, which involves intestinal colonization, fimbrial adhesion, and enterotoxin production. Therefore, the protective effects observed in this model should be interpreted with caution when extrapolating to enteric colibacillosis in calves. Further validation using intestinal infection models or naturally infected calves is required to confirm the protective efficacy of F1 under field-relevant conditions. The LD_50_ of K99-2 in mice was determined to be 6.9 × 10^7^ CFU, providing a dose basis for subsequent therapeutic experiments. During the treatment study, mice infected with K99-2 developed obvious clinical symptoms and showed a high mortality rate. In contrast, oral administration of F1 increased the survival rate of infected mice from 40% to 73%, indicating a protective effect of F1 against K99-2 infection.

The protective effect of F1 in infected mice may be associated with the combined actions of multiple constituents [54,55]. On the one hand, F1 treatment was associated with inhibition of K99-2 growth, alterations in bacterial cell-envelope integrity, membrane depolarization, and disruption of energy metabolism. On the other hand, some flavonoid and polyphenolic constituents detected in F1 may possess anti-inflammatory or immunomodulatory activities. However, whether these potential anti-inflammatory or immunomodulatory activities contribute to the protective effect of F1 during infection remains unclear and requires further experimental validation.

In conclusion, the compound herbal formula F1 obtained in this study exhibited antibacterial activity against *E. coli* K99 and was associated with multiple antibacterial effects, including alterations in cell wall and membrane integrity, membrane depolarization, intracellular component leakage, and disruption of ATP-related energy metabolism. Meanwhile, F1 showed favorable preliminary in vivo safety and increased the survival rate of K99-2-infected mice, indicating its potential for further investigation as a candidate veterinary herbal preparation.

This study provides experimental evidence supporting the potential of F1 as a candidate herbal preparation for further investigation against bovine-derived *E. coli* K99-associated infections. Future studies should focus on targeted quantitative analysis of the major active components of F1, validation of key bioactive compounds, pharmacokinetic characteristics, long-term safety, and evaluation of therapeutic efficacy in naturally infected calves. Moreover, the relatively high dose used in the mouse protection assay should be considered when translating F1 to practical veterinary applications. Direct extrapolation of the mouse dose to neonatal calves may not be feasible due to potential limitations in administration volume, palatability, and gastrointestinal tolerance. Therefore, further studies involving dose optimization, formulation improvement, and evaluation in naturally infected calves are required to further assess its practical application potential. These efforts will facilitate the translation of F1 from experimental research to veterinary application and provide further evidence for the development of Chinese herbal medicine-based strategies for controlling bacterial diarrhea in cattle.

## 5. Conclusions

In this study, compound herbal formula F1 was systematically evaluated from multiple aspects, including in vitro antibacterial activity, chemical composition analysis, antibacterial mechanisms, safety assessment, and therapeutic efficacy against infection. The results demonstrated that F1 exhibited antibacterial activity against the K99-2 strain. LC-MS analysis revealed multiple classes of candidate compounds, including furan derivatives, amino acids, flavonoids, galloylated polyphenols, and organic acids.

Mechanistic studies revealed that F1 exerted antibacterial effects associated with multiple cellular processes, including alterations in bacterial cell wall and membrane integrity, induction of membrane depolarization, leakage of intracellular components, and disruption of ATP-related energy metabolism. Notably, its antibacterial activity was not dependent on direct interaction with bacterial genomic DNA.

Animal experiments further demonstrated that F1 showed preliminary favorable short-term oral safety under the tested conditions within the tested dose range and improved the 7-day survival rate of K99-2-infected mice from 40% to 73%, indicating a protective effect in vivo.

Collectively, compound herbal formula F1 exhibits promising potential as a candidate herbal preparation for the prevention and control of *E. coli* K99 infection. Further studies focusing on the identification and quantification of its major active constituents, in vivo mechanisms, pharmacokinetic characteristics, long-term safety, and therapeutic efficacy in naturally infected calves are required to validate its translational potential.

## Figures and Tables

**Table 1:** Chinese medicinal materials, Latin names, and medicinal parts.

No.	Chinese Medicinal Material	Latin Name	Medicinal Part	No.	Chinese Medicinal Material	Latin Name	Medicinal Part
1	Herba Euphorbiae Humifusae	*Euphorbia humifusa* Willd.	Whole herb	36	Chinese gallnut	*Galla chinensis*	Insect gall
2	Guanhuangbai	*Phellodendron amurense* Rupr.	Stem bark	37	Acanthopanax bark	*Acanthopanax gracilistylus* W.W.Sm.	Root bark
3	Fennel	*Foeniculum vulgare* Mill.	Fruit	38	Ash bark	*Fraxinus rhynchophylla* Hance	Stem bark
4	Common self-heal spike	*Prunella vulgaris* L.	Fruit spike	39	Fortune eupatorium herb	*Eupatorium fortunei* Turcz.	Aerial parts
5	Houttuynia cordata	*Houttuynia cordata* Thunb.	Whole herb	40	Rhubarb	*Rheum palmatum* L.	Root and rhizome
6	Garden burnet root	*Sanguisorba officinalis* L.	Root	41	Artemisia capillaris herb	*Artemisia capillaris* Thunb.	Aerial parts
7	Glossy privet fruit	*Ligustrum lucidum* Aiton	Mature fruit	42	Sargentodoxa stem	*Sargentodoxa cuneata* (Oliv.) Rehder et E.H.Wilson	Stem
8	Sappan wood	*Caesalpinia sappan* L.	Heartwood	43	Lonicera flower bud	*Lonicera macranthoides* Hand.-Mazz.	Flower bud
9	Andrographis herb	*Andrographis paniculata* (Burm.f.) Nees	Whole herb	44	Astragalus root	*Astragalus membranaceus* (Fisch.) Bunge	Root
10	Chinese thorowax root	*Bupleurum chinense* DC.	Root	45	Isatis leaf	*Isatis indigotica* Fortune	Leaf
11	Violet herb	*Viola yedoensis* Makino	Whole herb	46	Angelica root	*Angelica sinensis* (Oliv.) Diels	Root
12	Lycium root bark	*Lycium chinense* Mill.	Root bark	47	Rhodiola root and rhizome	*Rhodiola rosea* L.	Root and rhizome
13	Cinnamon bark	*Cinnamomum cassia* (L.) J. Presl	Stem bark	48	Coptis rhizome	*Coptis chinensis* Franch.	Rhizome
14	Huangbai (Chuanhuangbai)	*Phellodendron chinense* Schneid.	Stem bark	49	Mugwort leaf	*Artemisia argyi* H. Lév. et Vaniot	Leaf
15	American ginseng	*Panax quinquefolius* L.	dried root	50	Sweet wormwood herb	*Artemisia annua* L.	Whole herb
16	Aucklandia root	*Saussurea costus* (Falc.) Lipsch.	Root	51	Saposhnikovia root	*Saposhnikovia divaricata* (Turcz.) Schischk.	Root
17	Blackberry lily rhizome	*Belamcanda chinensis* (L.) Redouté	Rhizome	52	Patrinia herb	*Patrinia scabiosaefolia* Fisch. ex Trevir.	Whole herb
18	Rose myrtle	*Rhodomyrtus tomentosa* (Aiton) Hassk.	Root/Fruit	53	Amomum fruit	*Amomum villosum* Lour.	Fruit
19	Milkwort root	*Polygala tenuifolia* Willd.	Root	54	Perilla leaf	*Perilla frutescens* (L.) Britton	Leaf
20	Magnolia bark	*Magnolia officinalis* Rehd. et Wils.	Stem bark	55	Gentiana root	*Gentiana macrophylla* Pall.	Root
21	Eucommia bark	*Eucommia ulmoides* Oliv.	Stem bark	56	Three-needle root	*Berberis soulieana* C.K. Schneid.	Root
22	Clove	*Syzygium aromaticum* (L.) Merr. et L.M. Perry	Flower bud	57	Gelsemium herb	*Gelsemium elegans* (Gardner et Champ.) Benth.	Whole herb (toxic)
23	Chinese pulsatilla root	*Pulsatilla chinensis* (Bunge) Regel	Root	58	Cinnamon twig	*Cinnamomum cassia* (L.) J. Presl	Young branch
24	Purple coneflower	*Echinacea purpurea* (L.) Moench	Aerial parts/Root	59	Stephania root	*Stephania japonica* (Thunb.) Miers	Root
25	Japanese knotweed rhizome	*Reynoutria japonica* Houtt.	Rhizome and root	60	Atractylodes rhizome	*Atractylodes macrocephala* Koidz.	Rhizome
26	Agastache herb	*Agastache rugosa* (Fisch. et C.A. Mey.) Kuntze	Aerial parts	61	Codonopsis root	*Codonopsis pilosula* (Franch.) Nannf.	Root
27	Areca peel	*Areca catechu* L.	Fruit pericarp	62	Kudzu root	*Pueraria lobata* (Willd.) Ohwi	Root
28	Charred hawthorn	*Crataegus pinnatifida* Bunge	Fruit (processed by stir-frying)	63	Coix seed	*Coix lacryma-jobi* L.	Seed kernel
29	Perilla stem	*Perilla frutescens* (L.) Britton	Stem	64	Licorice	*Glycyrrhiza uralensis* Fisch. ex. DC.	Root and rhizome
30	Cornus fruit pulp	*Cornus officinalis* Siebold et Zucc.	Fruit pulp	65	Finger citron	*Citrus medica* L. var. sarcodactylis Swingle	Fruit
31	Charred malt	*Hordeum vulgare* L.	Fruit (processed by stir-frying)	66	Dandelion	*Taraxacum mongolicum* Hand. -Mazz.	Whole herb
32	Chebulic myrobalan	*Terminalia chebula* Retz.	Fruit	67	Forsythia fruit	*Forsythia suspensa* (Thunb.) Vahl	Fruit
33	Mint herb	*Mentha canadensis* L.	Aerial parts	68	Atractylodes lancea rhizome	*Atractylodes lancea* (Thunb.) DC.	Rhizome
34	Verbena herb	*Verbena officinalis* L.	Whole herb	69	Smoked plum	*Prunus mume* Siebold et Zucc.	Fruit (smoked)
35	Star anise	*Illicium verum* Hook.f.	Fruit	70	Isatis root	*Isatis indigotica* Fortune	Root

**Table 2:** Composition of the Chinese herbal compound formulations.

Herbal Medicine	Compound F1 (g)	Compound F2 (g)	Compound F3 (g)
Cornus fruit pulp	25	–	25
Smoked plum	25	25	15
Chebulic myrobalan	15	20	20
Charred hawthorn	20	20	20
Atractylodes rhizome	20	20	15
Sappan wood	12	15	–
Aucklandia root	10	10	6
Licorice	5	–	5
Chinese yam	–	20	–
Forsythia fruit	–	10	–
Garden burnet root	–	–	12

Note: “–” indicates that the corresponding herbal medicine was not included in the formulation.

**Table 3:** In vitro antibacterial effects of different Chinese herbal medicines against *E. coli* K99-2.

Chinese Medicine	Inhibition Zone Diameter (mm)	Sensitivity	Chinese Medicine	Inhibition Zone Diameter (mm)	Sensitivity
Euphorbia humifusa	8	Low sensitivity	Galla chinensis	–	–
Phellodendron bark	–	–	Acanthopanax bark	–	–
Fennel	–	–	Fraxinus bark	10	Low sensitivity
Prunella spike	–	–	Eupatorium herb	–	–
Houttuynia herb	–	–	Rhubarb	–	–
Sanguisorba root	11	Moderate sensitivity	Artemisia capillaris herb	–	–
Ligustrum fruit	8	Low sensitivity	Sargentodoxa stem	–	–
Sappan wood	16	High sensitivity	Honeysuckle flower	–	–
Andrographis herb	–	–	Astragalus root	–	–
Bupleurum root	–	–	Isatis leaf	–	–
Viola herb	8	Low sensitivity	Angelica root	–	–
Lycium root bark	8	Low sensitivity	Rhodiola root and rhizome	8	Low sensitivity
Cinnamon bark	–	–	Coptis rhizome	–	–
Honeysuckle flower bud	–	–	Artemisia leaf	–	–
Aucklandia root	–	–	Artemisia annua herb	–	–
Iris rhizome	–	–	Saposhnikovia root	–	–
Phellodendron bark	–	–	Patrinia herb	10	Low sensitivity
Rhodomyrtus root/fruit	–	–	Amomum fruit	–	–
Polygala root	–	–	Perilla leaf	–	–
Magnolia bark	10	Low sensitivity	Gentiana root	–	–
Eucommia bark	–	–	Berberis root	–	–
Clove bud	10	Low sensitivity	Gelsemium herb	–	–
Pulsatilla root	–	–	Cassia twig	–	–
Echinacea herb/root	–	–	Stephania root	–	–
Reynoutria rhizome and root	–	–	Atractylodes rhizome	–	–
Agastache herb	–	–	Codonopsis root	–	–
Areca pericarp	–	–	Pueraria root	–	–
Crataegus fruit (charred)	18	High sensitivity	Coix seed	–	–
Perilla stem	–	–	Licorice root and rhizome	–	–
Cornus fruit pulp	19	High sensitivity	Finger citron fruit	12	Moderate sensitivity
Barley fruit (charred)	–	–	Dandelion herb	11	Moderate sensitivity
Terminalia fruit	16	High sensitivity	Forsythia fruit	11	Moderate sensitivity
Mint herb	9	Low sensitivity	Atractylodes rhizome	–	–
Verbena herb	9	Low sensitivity	Mume fruit (smoked)	20	High sensitivity
Star anise fruit	–	–	Isatis root	–	–

Note: “–” indicates no antibacterial activity.

**Table 4:** In vitro antibacterial effects of different Chinese herbal compound formulas against *E. coli* K99-2.

Chinese Herbal Compound Formula	Inhibition Zone Diameter (mm)	Sensitivity
Compound formula 1	21	Extremely sensitive
Compound formula 2	19	Highly sensitive
Compound formula 3	19	Highly sensitive

**Table 5:** Minimum inhibitory concentrations and minimum bactericidal concentrations of different Chinese herbal compound formulas against *E. coli* K99-2.

Chinese Herbal Compound Formula	Minimum Inhibitory Concentration (MIC, mg/mL)	Minimum Bactericidal Concentration (MBC, mg/mL)
Chinese herbal compound F1	62.5 ± 0.0	62.5 ± 0.0
Chinese herbal compound F2	125.0 ± 0.0	125.0 ± 0.0
Chinese herbal compound F3	125.0 ± 0.0	125.0 ± 0.0

Note: Data are expressed as mean ± SD. The three independent determinations yielded identical endpoint concentrations.

**Table 6:** Top five candidate compounds identified in Chinese herbal compound formula F1 by LC–MS.

(**A**) Chromatographic and identification parameters							
**Rank**	**Compound Name**	**Formula**	**Rt (min)**	**Obs. *m*/*z***	**Adduct**	**Error (ppm)**	**mzCloud (%)**
1	5-Hydroxymethyl-2-furaldehyde	C_6_H_6_O_3_	2.740	127.03904	[M+H]^+^	1.19	93.4
2	DL-Arginine	C_6_H_14_N_4_O_2_	0.852	175.11888	[M+H]^+^	−0.42	80.0
3	Isoliquiritigenin	C_15_H_12_O_4_	8.898	257.08035	[M+H]^+^	−1.88	98.4
4	1,6-Bis-O-(3,4,5-trihydroxybenzoyl) hexopyranose	C_20_H_20_O_14_	6.039	483.07779	[M−H]^−^	−0.49	82.3
5	D- (−)-Quinic acid	C_7_H_12_O_6_	6.960	191.05525	[M−H]^−^	−4.47	96.6
(**B**) MS/MS fragments, peak area, and compound category							
**Rank**	**Compound Name**	**Major MS/MS Ions (*m*/*z*)**			**Peak Area**	**Category**	
1	5-Hydroxymethyl-2-furaldehyde	109.0284, 81.0338, 53.0393			8.05 × 10^9^	Furan derivative/carbohydrate degradation product	
2	DL-Arginine	70.0657, 60.0563, 116.0705, 130.0972			4.24 × 10^9^	Amino acid	
3	Isoliquiritigenin	137.0230, 147.0437, 119.0490			3.57 × 10^9^	Flavonoid/chalcone	
4	1,6-Bis-O-(3,4,5-trihydroxybenzoyl) hexopyranose	169.0131, 125.0231, 211.0242, 271.0460			2.96 × 10^9^	Galloylated polyphenol	
5	D- (−)-Quinic acid	85.0279, 93.0331, 127.0387			5.76 × 10^8^	Organic acid	

Note: The superscript “+” and “−” indicate positive- and negative-ion modes, respectively; [M+H]^+^ and [M−H]^−^ represent protonated and deprotonated molecular ions, respectively. Candidate compounds were screened using |ΔMass| < 5 ppm and an mzCloud spectral matching confidence ≥ 80%. Peak areas represent semi-quantitative MS responses rather than absolute compound concentrations. Compound annotations were not confirmed using authentic analytical standards.

**Table 7:** Clinical observation scores of mice after oral administration of Chinese herbal compound F1.

Observation Category	Observation Item	Clinical Criteria	Clinical Score
Survival status	Number of deaths, moribund state	No death or moribund state	0
Mental status	Mental condition, responsiveness	Energetic and responsive	0
Behavioral activity	Activity level, presence of abnormal behavior	Normal activity and no abnormal behavior	0
External appearance	Hair luster, skin integrity, ocular and nasal secretions	Smooth hair, normal skin and mucosa, no ocular or nasal secretions	0
Food and water intake	Food consumption, water intake, feeding behavior	Normal food and water intake	0
Fecal characteristics	Fecal consistency, color, presence of bloody stool/diarrhea	Formed feces, normal color, no bloody stool or diarrhea	0
Continuous body weight gain	Body weight growth trend	Stable weight gain	0

Note: Clinical signs were scored using a semi-quantitative scoring system: 0 = no abnormality, 1 = mild abnormality, and 2 = severe abnormality. All animals showed no abnormal clinical signs during the 28-day administration period.

**Table 8:** Blood biochemical parameters of mice in different experimental groups.

Group	WBC (10^9^/L)	RBC (10^12^/L)	HGB (g/L)	MCV (fL)	PLT (10^9^/L)	MCH (pg)
A	3.1 ± 0.54	9.93 ± 0.59	179 ± 10.03	59.23 ± 0.34	522.33 ± 22.4	18.03 ± 0.37
B	2.67 ± 0.87	10.18 ± 0.42	183.33 ± 5.44	57.1 ± 1.84	502.33 ± 15.8	18 ± 0.22
C	3.07 ± 1.59	10.16 ± 0.32	182 ± 2.83	58.03 ± 0.31	530.33 ± 54.32	17.97 ± 0.82
D	3.83 ± 1.07	10.22 ± 0.55	189.67 ± 10.87	58.43 ± 1.39	562.67 ± 67.51	18.57 ± 1.03
E	4.5 ± 1.71	9.94 ± 0.05	175.33 ± 4.92	56.87 ± 0.61	511.67 ± 45.64	17.63 ± 0.54
F	3 ± 0.90	9.79 ± 0.11	178 ± 3.56	58.23 ± 1.15	528.67 ± 9.74	18.2 ± 0.59

Note: Values are presented as mean ± SD (n = 5). Statistical analysis was performed using one-way analysis of variance (ANOVA) followed by Dunnett’s multiple comparisons test. No significant differences were observed among groups (*p* > 0.05).

**Table 9:** (**A**) Absolute organ weights of mice administered compound herbal formula F1 by gavage. (**B**) Relative organ indices of mice administered compound herbal formula F1 by gavage.

(**A**)					
**Group**	**Heart Weight (g)**	**Liver Weight (g)**	**Spleen Weight (g)**	**Lung Weight (g)**	**Kidney Weight (g)**
62.5 mg/mL	0.17 ± 0.03 ^a^	1.40 ± 0.23 ^a^	0.08 ± 0.02 ^a^	0.23 ± 0.01 ^a^	0.40 ± 0.03 ^a^
125 mg/mL	0.19 ± 0.02 ^a^	1.50 ± 0.16 ^a^	0.07 ± 0.02 ^b^	0.21 ± 0.01 ^a^	0.41 ± 0.02 ^a^
250 mg/mL	0.16 ± 0.01 ^a^	1.55 ± 0.13 ^a^	0.10 ± 0.01 ^a^	0.17 ± 0.02 ^b^	0.40 ± 0.02 ^a^
600 mg/mL	0.16 ± 0.02 ^a^	1.47 ± 0.15 ^a^	0.08 ± 0.01 ^a^	0.21 ± 0.03 ^a^	0.37 ± 0.02 ^b^
1000 mg/mL	0.19 ± 0.02 ^a^	1.74 ± 0.17 ^a^	0.12 ± 0.03 ^a^	0.27 ± 0.04 ^a^	0.48 ± 0.04 ^a^
PBS	0.19 ± 0.02 ^a^	1.66 ± 0.21 ^a^	0.13 ± 0.06 ^a^	0.26 ± 0.04 ^a^	0.44 ± 0.03 ^a^
(**B**)					
**Group**	**Heart Index (%)**	**Liver Index (%)**	**Spleen Index (%)**	**Lung Index (%)**	**Kidney Index (%)**
62.5 mg/mL	0.48 ± 0.11 ^a^	3.95 ± 0.48 ^a^	0.22 ± 0.07 ^a^	0.65 ± 0.09 ^a^	1.13 ± 0.15 ^a^
125 mg/mL	0.53 ± 0.05 ^a^	4.20 ± 0.35 ^a^	0.20 ± 0.06 ^b^	0.60 ± 0.07 ^a^	1.14 ± 0.10 ^a^
250 mg/mL	0.45 ± 0.04 ^a^	4.38 ± 0.42 ^a^	0.28 ± 0.04 ^a^	0.49 ± 0.06 ^b^	1.13 ± 0.08 ^a^
600 mg/mL	0.49 ± 0.06 ^a^	4.36 ± 0.42 ^a^	0.23 ± 0.03 ^a^	0.63 ± 0.08 ^a^	1.09 ± 0.08 ^b^
1000 mg/mL	0.50 ± 0.06 ^a^	4.68 ± 0.37 ^a^	0.31 ± 0.08 ^a^	0.72 ± 0.12 ^a^	1.30 ± 0.06 ^a^
PBS	0.56 ± 0.08 ^a^	4.83 ± 0.73 ^a^	0.38 ± 0.18 ^a^	0.74 ± 0.10 ^a^	1.26 ± 0.09 ^a^

Note: Values are presented as mean ± SD. Organ indices were calculated as organ weight/body weight × 100. Statistical analysis was performed using one-way ANOVA followed by Dunnett’s multiple comparisons test compared with the PBS control group. Different superscript letters indicate significant differences among groups (*p* < 0.05).

**Table 10:** LD_50_ of K99-2.

Strain	Inoculation Dose (CFU/Mouse)	No. of Deaths/Total No. of Mice	Median Lethal Dose (LD_50_, CFU)
K99-2	5.5 × 10^8^	5/5	6.9 × 10^7^
	5.5 × 10^7^	2/5	
	5.5 × 10^6^	0/5	
	5.5 × 10^5^	0/5	
	5.5 × 10^4^	0/5	

## Data Availability

Data will be made available on request.

## Abbreviations

Abbreviation	Full term
AKP	Alkaline phosphatase
BCA	Bicinchoninic acid
CHMs	Chinese herbal medicines
DDA-MS/MS	Data-dependent acquisition tandem mass spectrometry
ESI	Electrospray ionization
HGB	Hemoglobin
LB	Luria–Bertani
LD_50_	Median lethal dose
MFI	Mean fluorescence intensity
MIC	Minimum inhibitory concentration
PBS	Phosphate-buffered saline
QC	Quality control
SEM	Scanning electron microscopy
UPLC	Ultra-performance liquid chromatography
ATP	Adenosine triphosphate
CFU	Colony-forming units
CLSI	Clinical and Laboratory Standards Institute
DNA	Deoxyribonucleic acid
H&E	Hematoxylin and eosin
HPLC-MS	High-performance liquid chromatography–mass spectrometry
LC-MS	Liquid chromatography–mass spectrometry
MBC	Minimum bactericidal concentration
MH	Mueller–Hinton
MS	Mass spectrometry
PLT	Platelet count
RBC	Red blood cell count
